# Efficient Scaling up EV‐AAVs Production via Cellular Nanoporation for Familial Hypercholesterolaemia Therapy

**DOI:** 10.1002/jev2.70186

**Published:** 2025-11-11

**Authors:** Yuting Yan, Yi You, Shuhong Ma, Hui Yi, Guangduo Chen, Jie Ni, Changyan Chen, Wenyu Ke, Lingying Li, Rui Bai, Yuqing Ran, Wenjing Lu, Min Zhu, Yongshuai Zhang, Jing Dai, Man Qi, Feng Lan, Andrew S. Lee, Ran Zhang, Xujie Liu, Zhaoyang Chen

**Affiliations:** ^1^ Key Laboratory of Pluripotent Stem Cells in Cardiac Repair and Regeneration, State Key Laboratory of Cardiovascular Disease, National Center for Cardiovascular Diseases Chinese Academy of Medical Sciences and Peking Union Medical College, Fuwai Hospital Beijing China; ^2^ State Key Laboratory of Cardiovascular Disease Chinese Academy of Medical Sciences, Fuwai Hospital Shenzhen China; ^3^ Department of Cardiology, Heart Center of Fujian Province Fujian Medical University Union Hospital, Fujian Medical University Heart Center Fuzhou Fujian China; ^4^ Department of Cardiovascular Surgery of the First Affiliated Hospital & Institute for Cardiovascular Science, Suzhou Medical College Soochow University Suzhou Jiangsu China; ^5^ School of Chemical Biology and Biotechnology Peking University Shenzhen Graduate School Shenzhen China; ^6^ Division of Pediatric Cardiology, Department of Pediatric Medicine The Seventh Medical Center of Chinese PLA General Hospital Beijing China; ^7^ Wenzhou Medical University Graduate School Wenzhou China; ^8^ Beijing Laboratory for Cardiovascular Precision Medicine, The Key Laboratory of Biomedical Engineering for Cardiovascular Disease Research Ministry of Education, Beijing Anzhen Hospital, Capital Medical University Beijing China; ^9^ Department of Cardiovascular Medicine Chinese PLA General Hospital & Chinese PLA Medical School Beijing China

**Keywords:** cellular nanoporation technology, extracellular vesicles‐encapsulated AAV, familial hypercholesterolaemia, LDLR

## Abstract

Adeno‐associated virus (AAV)–mediated gene therapies face critical clinical limitations, including immune‐mediated neutralization by pre‐existing antibodies and dose‐dependent hepatotoxicity. Extracellular vesicle‐encapsulated AAVs (EV‐AAVs) offer a promising solution by shielding AAVs from antibody recognition, yet existing production methods remain inefficient and impractical for clinical application. Here, we developed a cellular nanoporation (CNP) platform that enables scalable, high‐yield generation of EV‐AAVs, achieving an approximately 11‐fold increase in production efficiency compared with conventional methods. In LDLR‐deficient murine models with pre‐existing neutralizing antibodies (1:200), EV‐AAV‐LDLR at half the standard AAV dose robustly restored hepatic LDL receptor expression and attenuated atherosclerosis progression. Notably, EV‐AAV exhibited superior immune evasion capabilities, maintaining 2.3‐fold higher hepatic transduction efficiency than conventional AAV upon secondary dosing due to antibody shielding. Importantly, EV‐AAV therapy markedly reduced hepatotoxicity, with serum AST/ALT levels comparable to saline‐treated controls, thereby overcoming a critical safety barrier of high‐dose AAV treatment. These results demonstrate CNP as a clinically translatable platform for scalable EV‐AAV manufacturing, enabling effective multi‐dose regimens while overcoming key immunological and toxicity barriers in liver‐directed gene therapy for familial hypercholesterolaemia.

## 1 Introduction

Familial hypercholesterolaemia (FH) represents the most prevalent autosomal dominant disorder of lipid metabolism, with an estimated global prevalence of 1:200 to 1:500 individuals (Hu et al. 2020). This condition confers a life‐threatening risk of premature atherosclerotic cardiovascular disease (ASCVD), driven by lifelong exposure to elevated low‐density lipoprotein cholesterol (LDL‐C) levels. (Nordestgaard et al. 2013; Tada et al. 2023; Zhao et al. 2020). Genetically, FH is predominantly caused by loss‐of‐function mutations in LDLR (90%), APOB (5%), and gain‐of‐function variants in PCSK9 (1%), all disrupting hepatic clearance of LDL‐C (Abifadel and Boileau 2023). Current lipid‐lowering therapies, including statins and PCSK9 inhibitors, substantially reduce LDL‐C levels in heterozygous FH, but therapeutic efficacy in homozygous FH (HoFH) remains poor owing to severe or complete LDL receptor deficiency. In such patients, lipoprotein apheresis or even liver transplantation is often required (Catapano et al. 2016). Therefore, it is necessary to find a safe and effective treatment.

Gene therapy is emerging as a prospective therapy for many currently incurable diseases, and most of them use adeno‐associated virus (AAV) as a delivery vector (Hannah et al. 2023). AAV‐based gene therapies are currently approved for treating more than 80 diseases, such as inherited retinal diseases, haemophilia B, diabetes, spinal muscular atrophy, familial hypercholesterolaemia and so on (Penny et al. 2018; Rowe and Ciulla 2024; Symington et al. 2024; Mendell et al. 2017). Despite its success, AAV‐based gene therapy faces significant challenges related to both efficacy and safety (Shieh et al. 2020; Guillou et al. 2022; Lek et al. 2023). For example, a clinical trial for homozygous familial hypercholesterolaemia (HoFH) revealed that the transduction efficiency of AAV8 in human liver tissue was significantly compromised (NCT02651675). Furthermore, high systemic AAV doses trigger dose‐limiting toxicities, including hepatotoxicity (elevated AST/ALT), heart failure, pulmonary failure, kidney dysfunction and acute immune responses, culminating in fatal outcomes in recent trials (Ho et al. 2023; Arabi et al. 2022; Servais et al. 2023; Long et al. 2024).

To circumvent these limitations, strategies such as transient immunosuppression (utilizing corticosteroids, sirolimus, mycophenolate mofetil, calcineurin inhibitors or rituximab) (Boutin et al. 2010; Elmore et al. 2020; Rana et al. 2024), and capsid engineering to evade NAbs have been pursued (Tang et al. 2024; Liu et al. 2024). While immunosuppression can partially mitigate NAb effects, its efficacy is variable and carries inherent risks of adverse events (Vrellaku et al. 2024; Oh et al. 2019; Li et al. 2022; Youssef et al. 2016). Engineered capsids offer promise for immune evasion but fail to address the fundamental issue of dose‐dependent hepatotoxicity (Tang et al. 2024). These severe complications underscore the urgent need for new strategies to enhance the safety and efficacy of AAV‐based therapies.

Extracellular vesicles (EVs), particularly exosomes, offer a promising alternative delivery system owing to their intrinsic biocompatibility, physiological barrier penetration and reduced immunogenicity (Takakura et al. 2024). Recent studies demonstrate that when AAV are packaged using conventional approaches, EVs can naturally encapsulate the viral particles to generate EV‐encapsulated adeno‐associated virus (EV‐AAVs). These vesicles effectively shield AAV capsids from neutralizing antibodies, thereby enabling efficient transduction even in hosts with pre‐existing immunity, a major obstacle that has long limited the clinical application of AAV‐based gene therapies and now offers a promising solution for patients who were previously ineligible for treatment (Li et al. 2023; Kwak et al. 2023; Kovacs et al. 2021; Liu et al. 2021). Despite this potential, the clinical application of EV‐AAVs is constrained by the low yields achievable with current production methods. We previously developed a cellular nanoporation (CNP) method wherein transient nanopores are generated on source cell surfaces. This focal membrane disruption, coupled with localized heating from CNP, upregulates heat shock proteins (HSPs) and elevates intracellular Ca^2^⁺levels. These effects collectively enhance EV production by >50‐fold, establishing a novel strategy for scalable EV‐AAV manufacturing (Yang et al. 2020).

In this study, we utilized the CNP technology to optimize the production and encapsulation efficiency of EV‐AAVs, achieving a 11‐fold enhancement in yield compared to conventional methods. Additionally, we generated EV‐AAVs carrying human LDLR and demonstrated that, even in the presence of neutralizing antibodies (1:200), it retained excellent transduction efficiency to the liver. To evaluate therapeutic efficacy of EV‐AAVs, dose‐response studies in mice revealed that half‐dose EV‐AAVs achieved comparable hepatic transduction and therapeutic efficacy to full‐dose conventional AAV. We then employed LDLR^−/−^ mice as a model for HoFH. We observed significant improvements in atherosclerotic plaque burden and restored lipid homeostasis following half‐dose EV‐AAVs treatment, regardless of pre‐existing NAbs. Notably, while high‐dose AAV induced severe hepatotoxicity and cytokine storm, EV‐AAV at equivalent doses maintained serum transaminase levels within normal ranges.

Together, our findings highlight the transformative potential of the CNP platform for the efficient and scalable production of EV‐AAVs. By directly overcoming key barriers in vector manufacturing, this technology enables liver‐directed gene therapy at reduced dosing while mitigating toxicity risks. Importantly, we demonstrate its clinical viability in a homozygous FH model, where CNP‐generated EV‐AAVs achieved robust therapeutic efficacy with an improved safety margin, establishing a promising path toward next‐generation treatments for otherwise refractory genetic disorders.

## 2 Materials and Methods

### 2.1 Cell Culture

HEK293T cells and HepG2 cells were cultured in high‐glucose Dulbecco's Modified Eagle Medium (DMEM) (Gibco, USA), supplemented with 10% fetal bovine serum (FBS) (Gibco, USA). The cells were maintained at 37°C in a humidified incubator with 5% CO_2_. Cells were routinely passaged for subculturing when they reached approximately 80%–90% confluency using 0.25% trypsin‐EDTA (Gibco, USA).

### 2.2 EV‐AAVs and AAV Production

To produce EV‐AAV and AAV, HEK293T cells were plated uniformly on a chip within the CNP system. Using polyethyleneimine (PEI) (MedChemExpress, USA), the pDP9 plasmid and AAV9‐X plasmid were transfected into the cells. After transfection, electric stimulation was applied using the CNP system at 0, 12, 24, 48, and 56 h. The cell supernatant and cell pellets were collected 72 h post‐transfection.

The cell supernatants were subjected to sequential gradient centrifugation at 500 × *g* for 10 min and 2000 × *g* for 20 min to remove cells and cellular debris. The supernatant was then ultracentrifuged at 12,000 × *g* for 30 min to eliminate apoptotic bodies and large extracellular vesicles. The supernatant from the 12,000 × *g* centrifugation was centrifuged at 120,000 × *g* for 90 min using a Type 70Ti rotor in an Optima L‐90K ultracentrifuge (both from Beckman Coulter). The supernatant was discarded, and the resulting pellet contained crude EV‐AAV. The crude extract was further subjected to density gradient iodixanol (Sigma‐Aldrich, USA) centrifugation at 190,000 × *g* for 3 h to purify the EV‐AAV. The third and fourth fractions were collected, diluted with PBS, and subjected to ultracentrifugation at 120,000 × *g* for 120 min to obtain purified EV‐AAV (Li et al. 2023).

The cell pellets were subjected to repeated freeze‐thaw cycles for AAV production to fully lyse the cells. The lysate was centrifuged at 5500 rpm for 5 min, and the supernatant was collected. Solid sodium chloride was added to achieve a final concentration of 1 M, followed by dissolution and centrifugation at 12,000 rpm for 15 min to remove the salt (Servicebio, China). To precipitate AAV, 5× PEG8000 (Sigma‐Aldrich, USA) was added to the supernatant, and the mixture was incubated on ice for 1 h. After centrifugation at 11,000 rpm for 15 min, the supernatant was discarded. PBS was added to dissolve the precipitate, followed by treatment with 1 µg/mL of RNase (Thermo Scientific, USA) and DNase (Thermo Scientific, USA) for 30 min. Equal chloroform volumes were added, and the mixture was vortexed and centrifuged at 12,000 rpm for 5 min. The aqueous phase collected from the top layer contained the AAV particles. The crude extract was further subjected to density gradient iodixanol centrifugation at 190,000 × *g* for 3 h to purify the AAV. The 9‐12 fractions were collected, diluted with PBS, and subjected to ultracentrifugation at 120,000 × *g* for 120 min to obtain purified AAV.

Both EV‐AAV and AAV were subjected to DNase I treatment followed by purification using the High Pure Viral Nucleic Acid Kit (Roche, Switzerland) prior to RT‐qPCR analysis. According to the manufacturer's instructions, EV‐AAVs and AAV titres were determined using the AAVpro Titration Kit (for Real‐Time PCR) Ver. 2 (Takara, Japan). Briefly, AAV samples were diluted in concentrations, and real‐time PCR was performed to quantify the viral genome copy number. The primers provided in the kit were used to amplify the specific sequences of the AAV vector. The viral titre was calculated by comparing the Ct (threshold cycle) values obtained from the samples to a standard curve generated with known concentrations of AAV genomes.

### 2.3 Empty AAV9 Particles

The empty AAV9 particles were obtained as a commercially available standard from Vector Builder (Catalogue #:EAAV9‐b). As provided by the manufacturer's certificate of analysis, the concentration of total virus particles was determined using A280 UV spectrophotometry.

### 2.4 Western Blot

For protein extraction, EV, EV‐AAVs and AAV samples were lysed in RIPA buffer supplemented with protease inhibitors (Selleck, USA) on ice for 30 min. The lysates were centrifuged at 12,500 × *g* for 15 min at 4°C to remove cellular debris. The protein concentration of EV samples was measured using a BCA Protein Assay Kit (Thermo Scientific, USA). The AAVpro Titration Kit (for Real‐Time PCR) Ver. 2 (Takara, Japan) was used to determine the viral titre for AAV and EV‐AAVs titration. Equal amounts of EV‐AAVs and EVs samples were used for protein concentration determination with the BCA Protein Assay Kit.

For Western blot analysis, 15 µg of protein from EVs and EV‐AAVs samples, and 1 × 10^5^ viral genomes of EV‐AAVs and AAV samples, were separated by SDS‐PAGE. The proteins were then transferred to a Polyvinylidene Fluoride membrane. The membrane was blocked with 5% non‐fat milk in TBST for 1 h and incubated overnight at 4°C with primary antibodies: anti‐TSG101 (Abcam, USA), anti‐CD63 (Abcam, USA), anti‐CD81(Abcam, USA), anti‐ALIX (Proteintech, USA), anti‐Flot1 (Abcam, USA) and anti‐AAV (Abcam, USA). After washing with TBST, the membrane was incubated with secondary antibodies for 1 h at room temperature. Chemiluminescence reagents (specific brand/model to be added) were applied to visualize the immunoreactive bands. Band densities were analyzed using Image Lab software.

### 2.5 Mice

All animal experiments were conducted according to the guidelines outlined in the Guide for the Care and Use of Laboratory Animals (National Institutes of Health, NIH Publication No. 78‐23, 1996) and performed according to the protocols approved by Ethics Committee of Shenzhen Hospital of Fuwai Hospital (IRB2021BG049). All experimental mice were male, with Ldlr^−/−^ and C57BL/6J mice purchased from Gempharmatech (China).

### 2.6 In Vivo Imaging of Small Animals

C57BL/6J mice were intravenously injected with empty AAV9 particles (1.2e11 vp) and, 1 week later, re‐injected with an equal volume of saline, EV‐AAV‐Fluc (1.2e11 vg) or AAV‐Fluc (1.2e11 vg). Four weeks post‐injection, the mice underwent local hair removal, followed by administration of a luciferase substrate (Yeasen, China). After a 10‐min incubation period, bioluminescence imaging was performed using an in vivo imaging system. The bioluminescent signal was analyzed to assess the transduction efficacy.

### 2.7 AAV NAb Assay

Heat‐inactivated (56°C for 30 min) serum samples underwent serial dilution in DMEM (Gibco, USA) ranging from 1:10 to 1:2000. AAV9‐Fluc were diluted in DMEM to a multiplicity of infection (MOI) of 10,000. Equal volumes of diluted serum and AAV9‐Fluc were combined and incubated at 37°C for 1 h to facilitate antibody‐virus complex formation. Concurrently, HepG2 cells were seeded into 96‐well plates at a density of 3 × 10⁴ cells/well in 100 µL of complete growth medium (DMEM supplemented with 10% FBS and 1% penicillin/streptomycin) and cultured for 24 h. Following incubation, the serum‐AAV mixture (100 µL) was added to cell monolayers and incubated for 24 h at 37°C under 5% CO_2_. Supernatants were aspirated, cells were washed twice with PBS, and luciferase activity was quantified by adding 50 µL of D‐luciferin substrate (150 µg/mL; abcam, USA) followed by a 15 min incubation in the dark. Luminescence signals were measured using a microplate reader. Neutralization titres were defined as the reciprocal serum dilution achieving ≥50% reduction in transduction efficiency compared to positive controls (virus‐only wells) (Zhang et al. 2020).

### 2.8 Constructing and Therapy a Low‐titre Pre‐existing Immunity FH Mouse Model

Ldlr^−/−^ mice are fed a Western diet (Hfkbio, China) for 11 weeks. Afterward, a tail vein injection of empty AAV particles (6e11 vp) is administered, and neutralizing antibody levels are measured 1 week post‐injection using a serum sample. Subsequently, the mice are given either saline, AAV (1.2e12 vg), or EV‐AAVs (6e11 vg) by tail vein injection. At the sixth week, the mice are euthanized, and blood, liver and aorta tissues are collected for further analysis.

### 2.9 Histological Analysis and Staining

Mice were anaesthetized with pentobarbital (65 mg/kg, intraperitoneally). Following perfusion with PBS, the heart, liver and aorta tissues were harvested. The aortic tissue was fixed overnight in 4% paraformaldehyde, followed by Oil Red O staining. The aorta was longitudinally sectioned using an insect needle, and images were captured using a Zeiss stereo microscope. The aortic root and liver tissues were fixed overnight in 4% paraformaldehyde, embedded in OCT compound, and sectioned into 5 µm slices. These sections were stained with Oil Red O, Masson's trichrome, Sirius Red, H&E, LDLR, α‐SMA and F4/80 antibodies. ImageJ software was used to analyze the extent of the lesions.

### 2.10 Serum Biochemistry Analysis

Blood samples were collected via retro‐orbital puncture after an overnight fasting period. The blood was centrifuged at 3000 rpm for 15 min at 4°C to isolate the serum. The following biochemical parameters were measured using commercially available kits according to the manufacturer's instructions: Total Cholesterol (Nanjing Jiancheng Bioengineering Institute, China), Triglycerides (Nanjing Jiancheng Bioengineering Institute, China), Low‐Density Lipoprotein Cholesterol (Nanjing Jiancheng Bioengineering Institute, China), Aspartate Aminotransferase (Nanjing Jiancheng Bioengineering Institute, China) and Alanine Aminotransferase (Nanjing Jiancheng Bioengineering Institute, China). The enzyme‐linked assay was conducted using a microplate reader.

### 2.11 Statistical Analysis

All mice were randomly assigned to different experimental groups, and the experiments were conducted in a blinded manner. Statistical comparisons between two groups were performed using an unpaired two‐tailed Student's *t*‐test, while one‐way ANOVA was used for comparisons involving multiple groups. Post‐hoc pairwise comparisons were conducted using Tukey's multiple comparison test implemented in Prism software. A mixed‐effects ANOVA was employed for serial measurements, and post‐hoc pairwise comparisons were adjusted using Bonferroni. The aortic lesion area was quantified using ImageJ software and expressed as the percentage of the total area of the entire vessel. Statistical significance was defined as *p < 0.05, **p < 0.01, ***p < 0.001, ****p < 0.0001 and ns. denoted insignificant. All data are presented as mean values ± standard error of the mean (SEM).

## 3 Result

### 3.1 Conventional Production and Purification of EV‐AAVs

AAV production and subsequent encapsulation were achieved in HEK293T cells using a dual‐plasmid transfection system. Seventy‐two hours post‐transfection, cell culture supernatants were harvested and subjected to gradient ultracentrifugation to obtain crude EV‐AAVs. Using a recently reported iodixanol density gradient centrifugation technique, we separated the crude EV‐AAVs into 12 distinct fractions (Li et al. 2023). To determine the fraction in which EV‐AAVs was most enriched, we performed titre assays on each fraction and observed AAV distribution across all fractions (Figure S1A). Additionally, we subjected the AAV collected from the cell pellet to iodixanol gradient centrifugation and found that free AAV was predominantly enriched in fractions 9, 10, 11 and 12 (Figure S1B,C). Western blot analysis of the EV markers Alix and Flotillin across the fractions revealed that EV‐AAVs was primarily concentrated in fractions 3 and 4, consistent with previous reports (Figure S1D).

### 3.2 Boost EV‐AAV Production Using CNP

**FIGURE 1:**
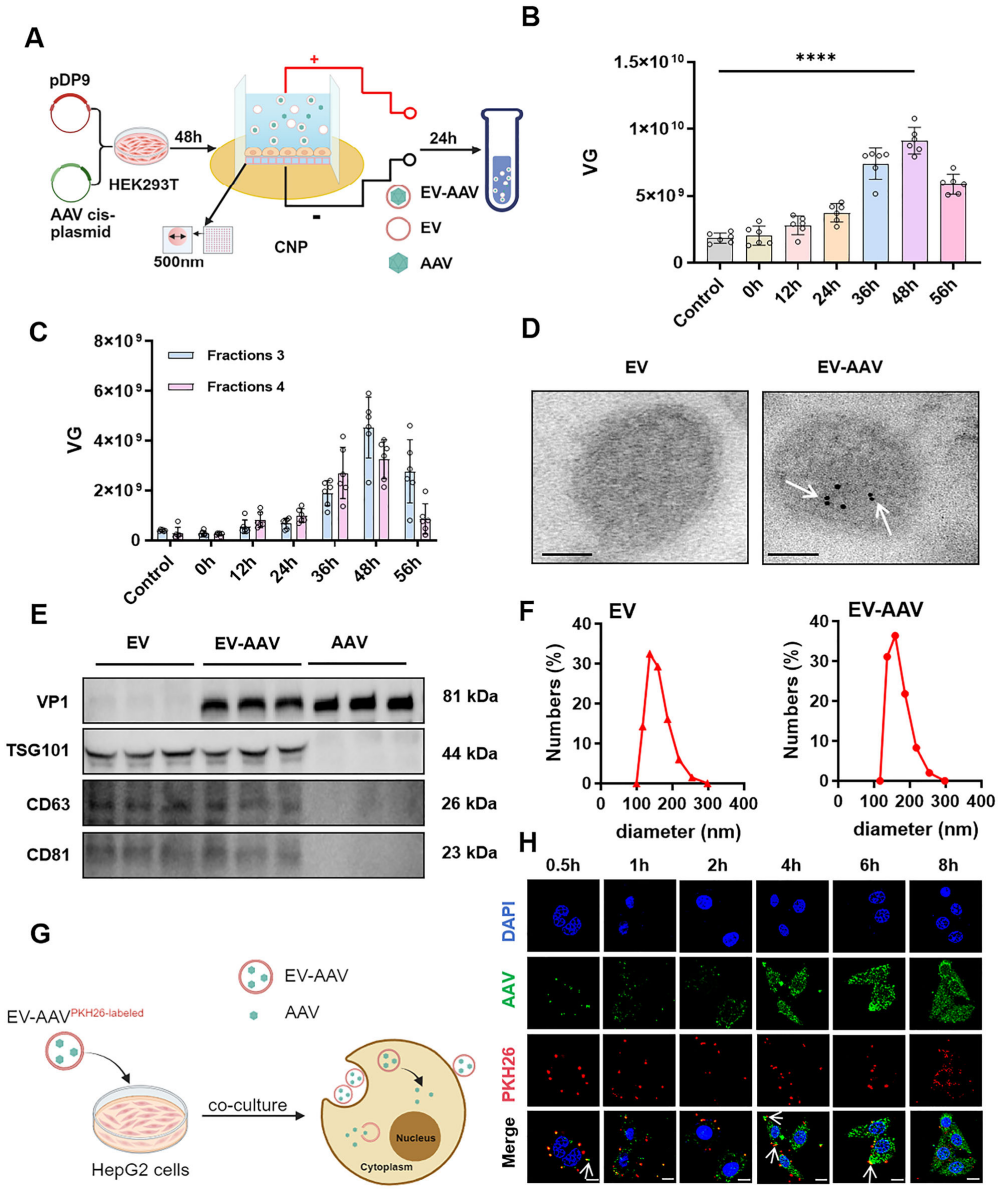
Optimization of EV‐AAV production using CNP. (A) Schematic illustration of EV‐AAVs packaging. (B) Total AAV titre in EV‐AAV after encapsulation without CNP stimulation or following CNP stimulation at 0, 12, 24, 48 and 56 h post‐encapsulation. Control: HEK293T cells harvested at 72 h without CNP treatment. *n* = 6, data were analyzed using two‐way ANOVA. Values are presented as mean ± SEM. ****p < 0.0001. (C) Titre analysis of third and fourth fractions of EV‐AAVs produced at different time points post‐iodixanol purification. (D) Transmission electron microscopy (TEM) and immunogold labelling images showing the morphological characteristics of purified EVs (left) and EV‐AAVs (right), AAV were labelled with antibodies coupled with gold particles (5 nm). White arrows indicate virus particles encapsulated by EVs. Scale bar = 50 nm. (E) Western blot analysis of TSG101, CD81, CD63 and VP1 protein content in EVs, EV‐AAVs and free AAV. (F) Particle size distribution of EVs and EV‐AAVs measured by ZetaView nanoparticle analyzer. (G) Schematic diagram of the intracellular uptake experiment for EV‐AAVs. (H) Fluorescence microscopy images showing the release of AAV from EV‐AAVs into HepG2 cells at various time points. PKH26 is labelled in red, AAV capsid protein is marked in green, and white arrows indicate the release of AAV from EV‐AAVs. Scale bar = 10 µm.

To enhance EV‐AAVs yield, we investigated the effect of CNP application timing by stimulating AAV‐encapsulating cells at 0, 12, 36, 48 and 56 h post‐transfection. At 72 h post‐transfection, the cell supernatants were collected, and crude EV‐AAVs were isolated using density gradient centrifugation (Figure 1A). We then measured the total titre of these crude EV‐AAVs preparations and found that CNP stimulation at 48 h resulted in the highest total yield, which was approximately 5‐fold greater than that of the control group (Figure 1B). To evaluate the yield of EV‐AAVs, we performed iodixanol density gradient centrifugation on EV‐AAVs harvested at different post‐CNP time points. Fractions 3 and 4 were subsequently collected for titre quantification. EV‐AAVs harvested at 48 h post‐CNP demonstrated the highest yield, reaching approximately 10.97‐fold higher levels compared to the control group (Figures 1C and S1E). This enhanced yield was accompanied by a significant increase in AAV encapsulation efficiency, which was also highest at 48 h and showed a 10.86‐fold increase as measured by the ratio of EV‐AAV titre to total AAV titre in the supernatant (Figure S1F). These findings, combined with our observation that CNP treatment induced an 11.25‐fold increase in total EV quantity (Figure S2A), suggesting that the elevated yield of EV‐AAV is mechanistically attributable to augmented EV production rather than enhanced viral packaging efficiency.

Next, to elucidate the underlying mechanisms of CNP‐boosted EV‐AAV yield, specifically whether it involves active endocytosis or passive entrapment, we pre‐treated cells with bafilomycin A1, an inhibitor of endosomal acidification. As bafilomycin A1 did not significantly suppress EV‐AAV titres after CNP treatment (Figure S2B), our results indicated that the enhanced production is driven by CNP‐triggered transient membrane disruption rather than an endocytic pathway. Further analysis revealed the molecular basis for this process. KEGG enrichment analysis revealed significant upregulation of the PI3K‐Akt signalling and calcium signalling pathways after CNP treatment (Figure S2C). Additionally, a volcano plot analysis highlighted that CNP significantly upregulates membrane repair proteins (Figure S2D). Subsequent qPCR validation confirmed the marked induction of key membrane repair genes, including HSP90, HSP70, TP53INP1 and CHMP4BP1 (Figure S2E). These findings are consistent with prior studies, which demonstrate that calcium influx and HSP upregulation trigger rapid membrane repair and EV shedding (Yang et al. 2020). Based on these results, we propose that during this rapid membrane restructuring, AAVs are passively co‐encapsulated into newly formed EVs without requiring active endosomal recruitment.

To examine EV‐AAV generated by this method, transmission electron microscopy (TEM) and immunogold labelling revealed that intact AAV particles encapsulated within EV displaying a bilayer membrane (Figure 1D). Confocal microscopy confirmed these findings (Figure S3A). Western blot confirmed the presence of EV marker and AAV capsid proteins in purified EV‐AAV (Figure 1E). Additionally, EV‐AAV particle size was comparable to EVs, with no significant difference in zeta potential (Figures 1F and S3B). To evaluate the kinetics of AAV release from EV‐AAVs, we co‐cultured EV‐AAVs with HepG2 cells and monitored AAV release over a time course (0.5, 1, 2, 4, 6 and 8 h) using confocal microscopy (Figure 1G). In HepG2 cells, the intracellular AAV accumulation increased over time, while the number of intact EV‐AAV complexes progressively declined—peaking at 4 and 0.5 h post‐incubation, respectively. Notably, over 80% of the AAVs were efficiently packaged into EVs within the first 0.5 h (Figures 1H and S3C). These data establish that CNP‐based methodology significantly optimizes EV‐AAV production, synergistically enhancing AAV encapsulation efficiency via augmented EV yield. The resulting EV‐AAVs retain their native EV‐like morphological integrity and cellular uptake functionality, while achieving an order of magnitude amplification relative to controls.

### 3.3 Sustained High Transduction Efficiency of EV‐AAVs Upon Second Injection

**FIGURE 2:**
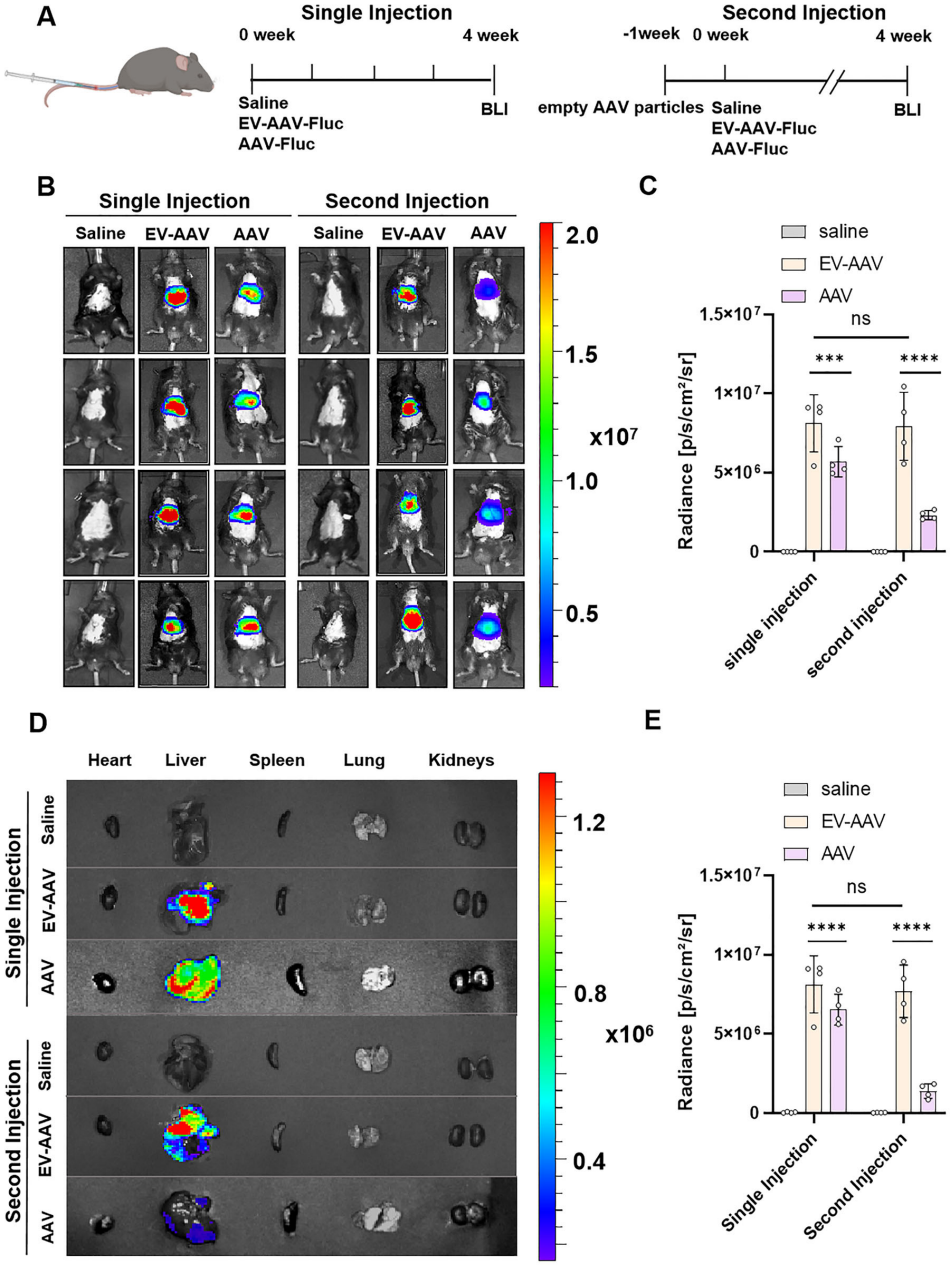
Sustained high transduction efficiency of EV‐AAV upon second injection. (A) Experimental design schematic. (B) In vivo bioluminescence imaging at 4 weeks post‐tail vein injection of saline (negative control), EV‐AAV9‐Fluc and AAV9‐Fluc (1.2e11 vg) (left). Pre‐injection of empty AAV9 particles (1.2e11 vp) followed by subsequent injections of saline, EV‐AAV9‐Fluc or AAV9‐Fluc (1.2e11 vg), with in vivo bioluminescence imaging taken at 4 weeks post‐injection (right). (C) Quantification of bioluminescence signals in the injection region of mice after single or double injections of saline, EV‐AAV9‐Fluc or AAV9‐Fluc at 4 weeks (*n* = 4). (D) Ex vivo imaging of heart, liver, spleen, lungs and kidneys from mice euthanized at 4 weeks post‐single or double injection of saline, EV‐AAV9‐Fluc or AAV9‐Fluc. (E) Quantification of bioluminescence signals in the injection region of mice after single or double injections of saline, EV‐AAV9‐Fluc or AAV9‐Fluc at 4 weeks (*n* = 4). For panels C and E, values were analyzed using two‐way ANOVA. Data are presented as mean ± SEM. ****p < 0.0001, ***p < 0.001, ns indicates no significant difference.

To assess the transduction efficiency of EV‐AAVs compared to AAV in vivo, we intravenously injected 5‐week‐old C57BL/6 mice with saline, EV‐AAV‐Fluc or AAV‐Fluc (1.2e11 vg). Additionally, a separate group of mice was pre‐injected with empty AAV particles, and neutralizing antibody titres were measured in their serum 7 days later. These mice were then re‐injected with saline, EV‐AAV‐Fluc or AAV‐Fluc (1.2e11 vg). Transduction efficiency was monitored in both single‐ and second‐injection cohorts via in vivo imaging at week 4 (Figure 2A).

In vivo, mice were administered either a single EV‐AAV‐Fluc injection or a prime injection of AAV empty capsids followed by EV‐AAV‐Fluc. Bioluminescence imaging revealed that EV‐AAV‐Fluc exhibited a 1.42‐fold increase in hepatic transduction efficiency compared to AAV‐Fluc. Notably, EV‐AAV‐Fluc retained efficacy upon repeated administration, whereas AAV‐Fluc efficacy dropped over 71%, reaching only 29% EV‐AAV‐Fluc singal (Figure 2B,C). Furthermore, ex vivo imaging revealed pronounced hepatic tropism for EV, EV‐AAV and unmodified AAV vectors. Notably, luciferase expression in the AAV‐Fluc group was markedly diminished after secondary injection, consistent with attenuated whole‐body bioluminescence patterns observed in vivo (Figures 2D,E and S4A,B).

### 3.4 Half Dose EV‐AAV‐Fluc Achieves Comparable Transduction to Full Dose AAV‐Fluc

**FIGURE 3:**
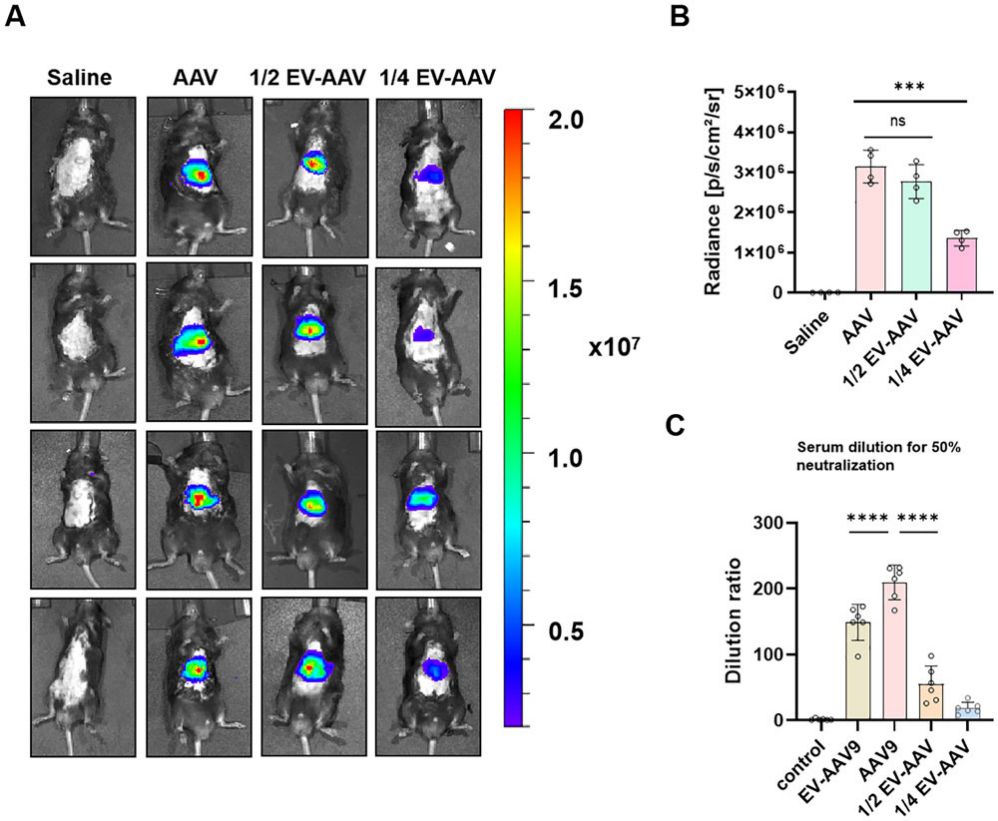
EV‐AAV‐FLUC at half the dose achieves comparable transduction efficiency to AAV‐FLUC. (A) Tail vein injection of saline, AAV (1.2e11 vg), EV‐AAVs (6e10 vg) and EV‐AAVs (3e10 vg) in C57 mice, followed by in vivo bioluminescence imaging of liver regions 1 week post‐injection. *n* = 4. (B) Quantification of bioluminescence signals in the liver regions of mice after tail vein injection of saline, AAV (1.2e11 vg), EV‐AAVs (6e10 vg) or EV‐AAVs (3e10 vg) 1 week post‐injection. (C) Measurement of neutralizing antibody levels in mouse serum 1 week after injection of saline, AAV (1.2e11 vg), EV‐AAVs (1.2e11 vg), EV‐AAVs (6e10 vg) or EV‐AAVs (3e10 vg). *n* = 6. For panels B and C, data were analyzed using two‐way ANOVA. Values are presented as mean ± SEM. ****p < 0.0001, ***p < 0.001, ns indicates no significant difference.

To explore the potential of lower‐dose EV‐AAV treatments, we compared the transduction efficiency of 1/2‐dose EV‐AAV‐Fluc (6e10 vg), 1/4‐dose EV‐AAV‐Fluc (3e10 vg) and AAV‐FLUC (1.2e11 vg) (Figure 3A). We found that mice receiving 1/2‐dose EV‐AAV‐Fluc exhibited luciferase expression comparable to that of mice administrated a full dose of AAV‐Fluc, with no significant difference in fluorescence intensity (Figure 3B). Furthermore, we observed that neutralizing antibody titres induced by EV‐AAV‐Fluc were lower than those generated by AAV‐Fluc, and antibody titres correlated positively with the injection dose (Figure 3C). Based on these findings, we selected the 1/2‐dose EV‐AAVs as the treatment dose for subsequent therapeutic experiments.

### 3.5 EV‐AAV‐LDLR Restores LDLR Expression and Improves Lipid Profile

**FIGURE 4:**
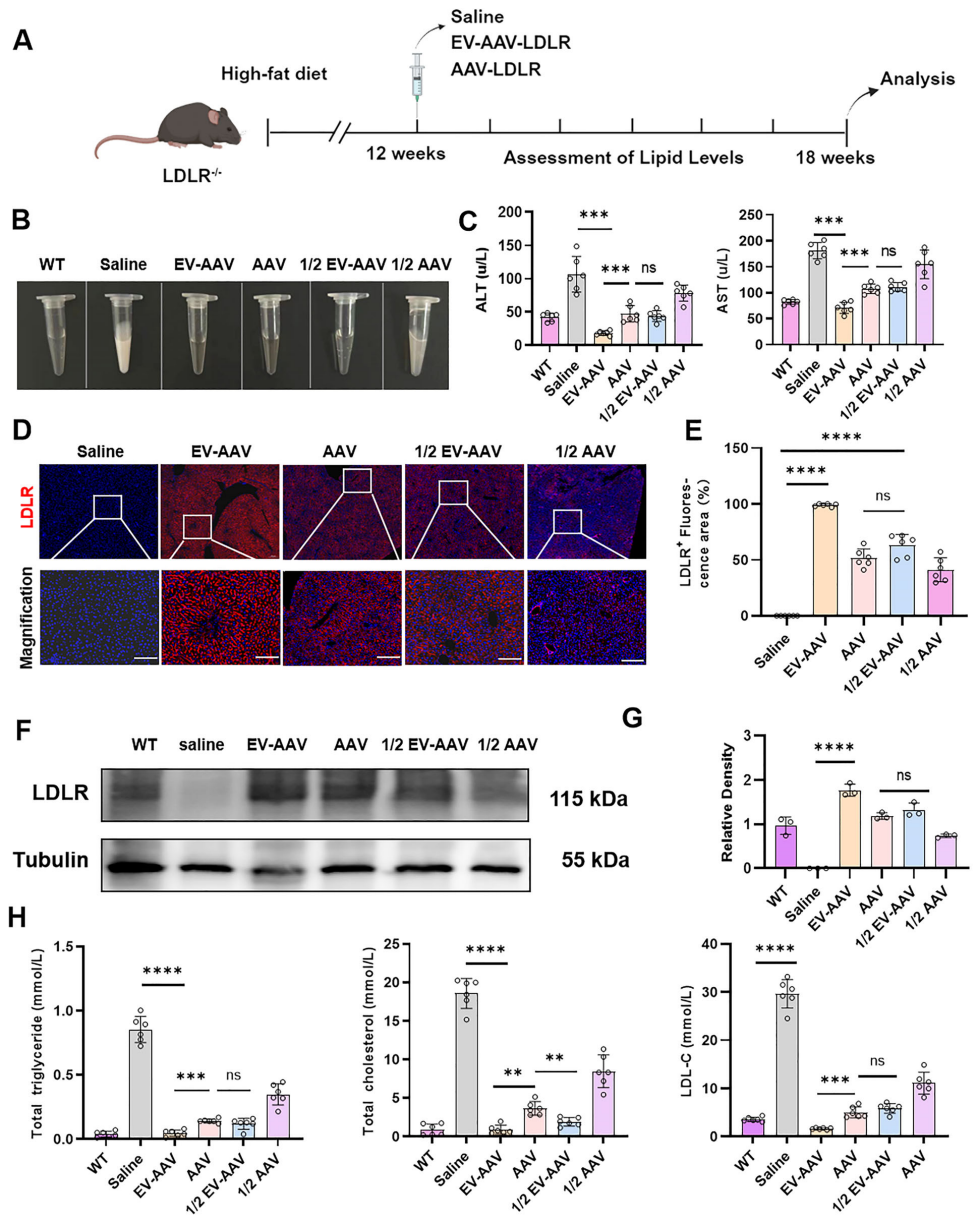
EV‐AAV9‐LDLR restores LDLR expression and improves lipid profile. (A) Experimental design. LDLR^−/−^ mice were fed either a Western diet for 12 weeks or not, followed by tail vein injection of saline, EV‐AAV‐LDLR (1.2e11 vg), AAV‐LDLR (1.2e11 vg), EV‐AAV‐LDLR (6e10 vg) or AAV‐LDLR (6e10 vg). Analysis was performed 6 weeks post‐injection. *n* = 6. (B) Morphological observation of mouse serum samples. (C) Analysis of serum AST and ALT levels in mice. (D) Immunofluorescence analysis of LDLR recovery in mouse liver tissue post‐euthanasia. Scale bar = 100 µm. (E) ImageJ analysis of LDLR fluorescence intensity. (F) Western blot detection of LDLR protein levels in mouse liver tissue. (G) Gray scale analysis of images from panel F using ImageJ. (H) Measurement of total cholesterol, triglycerides, and LDL‐C levels in mouse serum. For panels C, E, G and H, data were analyzed using two‐way ANOVA. Values are presented as mean ± SEM. ****p < 0.0001, ***p < 0.001, **p < 0.01, *p < 0.05, ns indicates no significant difference.

To evaluate the therapeutic potential of EV‐AAV‐LDLR, we fed 4‐week‐old LDLR^−/−^ mice a high‐fat diet for 12 weeks, followed by intravenous injection of saline, AAV‐LDLR (1.2e12 vg), EV‐AAV‐LDLR (1.2e12 vg) or EV‐AAV‐LDLR at half‐dose (6e11 vg). Blood samples were collected at 1, 2, 4 and 6 weeks post‐injection for lipid analysis, and mice were euthanized at week 6 for phenotypic evaluation (Figure 4A). At week 6, the saline group displayed pronounced chylomicronaemia, while all treatment groups exhibited clear amelioration of this phenotype (Figure 4B). In addition, full‐dose of EV‐AAV‐LDLR treatment significantly restored serum ALT and AST levels in LDLR^−/−^ mice to normal values (Figure 4C), suggesting a substantial recovery of liver function. Immunofluorescence staining further revealed that EV‐AAV‐LDLR treatment nearly restored LDLR expression in the livers of LDLR^−/−^ mice, with AAV‐LDLR and the 1/2‐dose EV‐AAV‐LDLR groups showing partial recovery (Figure 4D,E). Western blot analysis confirmed these results (Figure 4F,G), indicating a dose‐dependent effect of EV‐AAV‐LDLR treatment. Throughout the treatment, total cholesterol, triglycerides and low‐density lipoprotein (LDL) levels gradually returned to normal, with near‐complete recovery observed by week 6 (Figures S5A–C and 4H).

### 3.6 EV‐AAV‐LDLR Alleviates Atherosclerotic Progress in FH Mice at a Lower Dose

**FIGURE 5:**
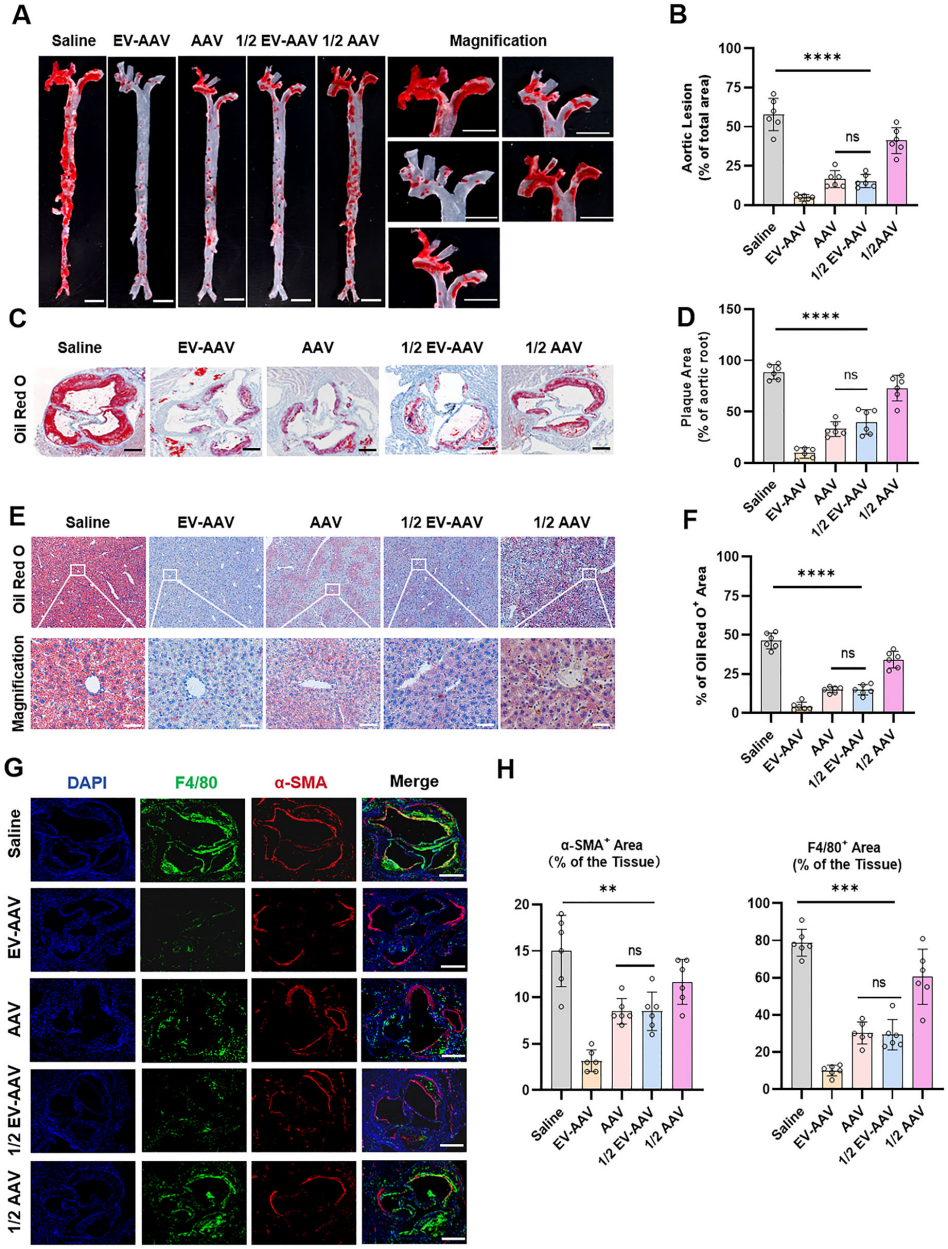
EV‐AAV‐LDLR improves atherosclerotic phenotype. (A) LDLR^−/−^ mice were fed a Western diet for 11 weeks or not, followed by tail vein injection of saline, EV‐AAV‐LDLR (1.2e11 vg), AAV‐LDLR (1.2e11 vg), EV‐AAV‐LDLR (6e10 vg) or AAV‐LDLR (6e10 vg). Mice were euthanized 6 weeks post‐injection, and aortic tissue was collected for Oil Red O staining to analyze lipid content. (B) Quantification of lipid content in aortic tissue from panel A using ImageJ. Scale bar = 5 mm (C) Representative images of Oil Red O staining in the aortic valve. Scale bar = 200 µm. (D) ImageJ analysis of lipid deposition in the aortic valve. (E) Representative images of Oil Red O staining in liver tissue. Scale bar = 50 µm. (F) Quantification of lipid deposition in liver tissue using ImageJ. (G) Immunofluorescence analysis of α‐SMA and F4/80 expression and distribution in the aortic valve. Scale bar = 200 µm. (H) ImageJ analysis of α‐SMA and F4/80 expression levels. n=6, data were analyzed using two‐way ANOVA. Values are presented as mean ± SEM. ****p < 0.0001, ***p < 0.00, **p < 0.01, ns indicates no significant difference.

To further assess the therapeutic efficacy of EV‐AAV‐LDLR, we evaluated the atherosclerotic phenotypes in LDLR^−/−^ mice treated with saline, EV‐AAV‐LDLR and AAV‐LDLR, half‐dose EV‐AAV‐LDLR and AAV‐LDLR. Examination of the aortic arch revealed a significant reduction in aortic lesion in the EV‐AAV‐LDLR treatment group (Figure S6A,B). Macroscopic Oil Red O staining of the aorta showed varying degrees of plaque reduction in the treatment groups, with EV‐AAV‐LDLR demonstrating near‐complete plaque regression compared to AAV‐LDLR and 1/2‐dose EV‐AAV‐LDLR (Figure 5A,B). Oil Red O staining of the aortic root revealed a significant reduction in staining in the EV‐AAV‐LDLR group. In contrast, therapeutic efficacy between the AAV‐LDLR and half‐dose EV‐AAV‐LDLR cohorts showed no statistically significant divergence (*p* > 0.05), while the half‐dose AAV group exhibited less restoration of LDLR function (Figure 5C,D). These results indicate that EV‐AAV‐LDLR significantly improves the atherosclerotic phenotype in LDLR^−/−^ mice.

Liver Oil Red O staining showed that the EV‐AAV‐LDLR treatment group had normalized lipid droplet levels. While AAV‐LDLR and 1/2‐dose EV‐AAV‐LDLR also showed a significant reduction, their effect was less pronounced than with the full‐dose EV‐AAV‐LDLR treatment (Figure 5E,F). Furthermore, immunofluorescence analysis revealed that in the saline group, both the macrophage marker F4/80 and the smooth muscle marker α‐SMA were highly accumulated in the aortic root. In contrast, EV‐AAV‐LDLR treatment significantly reduced F4/80 expression and diminished α‐SMA positive. The AAV‐LDLR and 1/2‐dose EV‐AAV‐LDLR groups also showed similar trends, although the reduction levels were less pronounced than with the full‐dose EV‐AAV‐LDLR treatment. (Figure 5G,H). Additionally, Sirius Red, H&E and Masson's trichrome staining results showed that EV‐AAV‐LDLR treatment significantly reduced the fibrosis in the aortic root, with clear signs of morphological recovery observed (Figure S6C). Correspondingly, Liver staining with Sirius Red, H&E and Masson's revealed reduced vacuolization and partial recovery of hepatic fibrosis after EV‐AAV‐LDLR treatment (Figure S6D).

These findings collectively demonstrate that EV‐AAV‐LDLR exhibits potent therapeutic effects in hypercholesterolaemic LDLR^−/−^ mice. Notably, low‐dose EV‐AAV‐LDLR can achieve a therapeutic efficacy comparable to that of full dose of AAV‐LDLR, suggesting a significant improvement in delivery efficiency.

### 3.7 Half Dose EV‐AAV Outperforms AAV in Repeated FH Treatment

**FIGURE 6:**
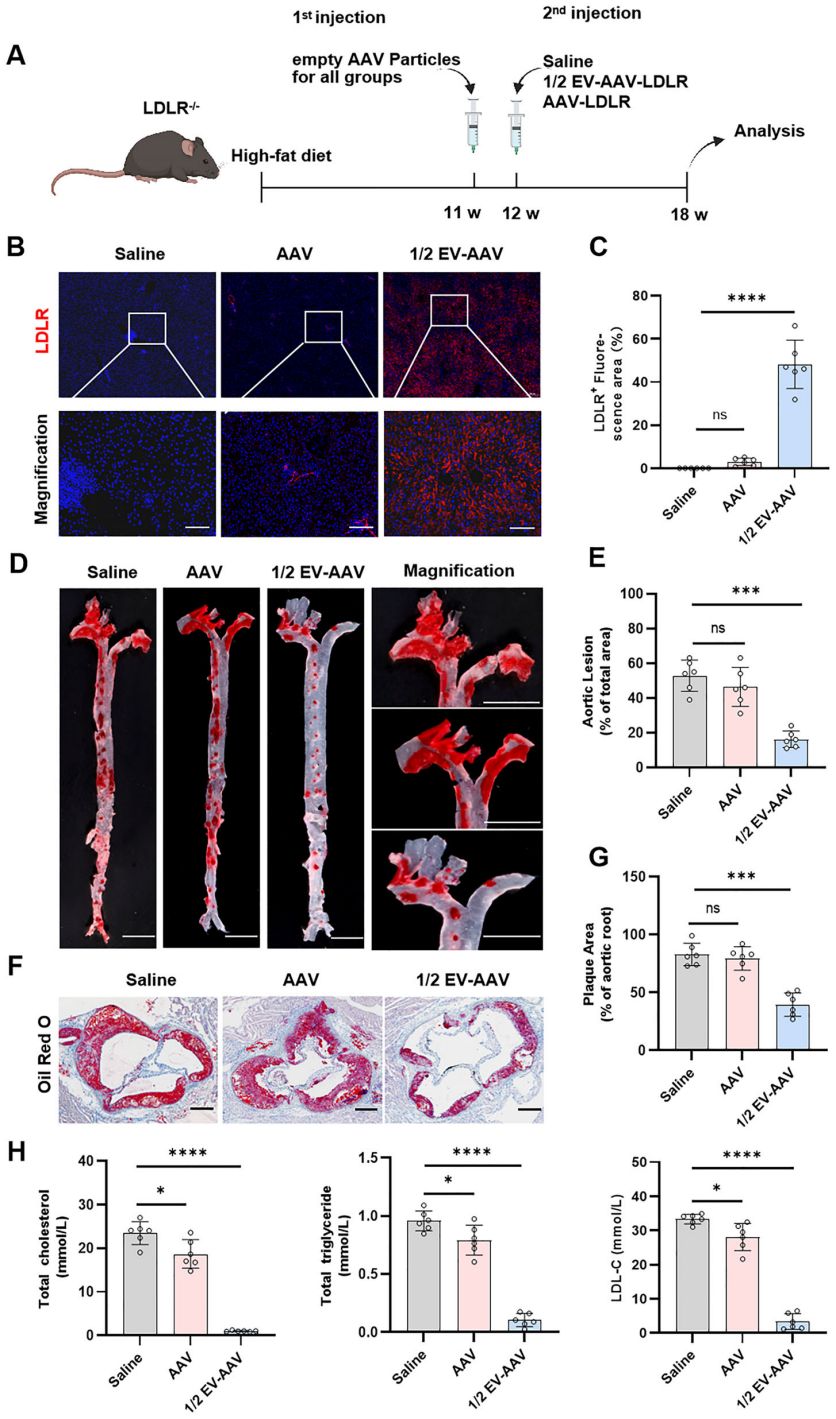
1/2 EV‐AAV‐LDLR maintains efficacy after repeated injection. (A) LDLR^−/−^ mice were fed a Western diet for 11 weeks, followed by tail vein injection of empty AAV9 particles (6e11 vp). After 1 week of continued Western diet feeding, mice were injected with saline, AAV‐LDLR (1.2e11 vg) or EV‐AAV‐LDLR (6e10 vg). Analysis was performed 6 weeks after injection. (B) Immunofluorescence analysis of LDLR expression in liver tissue from different groups of mice. Scale bar = 100 µm. (C) Quantification of LDLR expression levels in liver tissue using ImageJ. (D) Oil Red O staining to assess lipid deposition in the aorta. Scale bar = 5 mm. (E) Quantification of lipid content in the aorta using ImageJ. (F) Representative images of Oil Red O staining in the aortic valve. Scale bar = 200 µm. (G) ImageJ analysis of lipid deposition in the aortic valve. (H) Measurement of total cholesterol, triglycerides, and LDL‐C levels in serum. *n* = 6, data were analyzed using two‐way ANOVA. Values are presented as mean ± SEM. ****p < 0.0001, ***p < 0.001, *p < 0.05, ns indicates no significant difference.

We previously demonstrated that a half‐dose of EV‐AAV‐LDLR achieves therapeutic efficacy comparable to a full‐dose of AAV‐LDLR. However, it remains unclear whether low‐dose EV‐AAV‐LDLR retains its therapeutic effects after multiple injections. To investigate this, we first fed LDLR^−/−^ mice a Western‐style diet for 11 weeks. Following this, the mice received an initial intravenous injection of empty AAV9 particles. One week later, we measured neutralizing antibody titres and then administered a second intravenous injection of either saline injection, full‐dose AAV‐LDLR (1.2e12 vg), or half‐dose EV‐AAV‐LDLR (6e11 vg). Efficacy was evaluated 6 weeks post‐injection (Figure 6A). Notably, administration of AAV empty capsids induced neutralizing antibody titres reaching 1:200 within 1 week, thereby establishing a validated model of low‐titre pre‐existing immunity (Figure S7A).

Immunofluorescence analyses revealed significant restoration of LDLR expression in the 1/2 EV‐AAV‐LDLR group (Figure 6B,C). Furthermore, in the context of pre‐existing immunity, this group exhibited a significant reduction in plaque area at the aortic root compared to the saline control, while no notable changes were observed in the AAV group (Figure S7B,C).

Macroscopic and aortic root Oil Red O staining obtained similar results, with the 1/2‐dose EV‐AAV‐LDLR group exhibiting a marked reduction in both staining area and plaque number. In contrast, the AAV‐LDLR treatment exhibited only minimal improvement (Figure 6D–G). Oil Red O staining also showed reduced hepatic lipid droplets and improved lipid profiles in 1/2‐dose EV‐AAV‐LDLR group, whereas the AAV group showed limited LDLR restoration and less significant improvement (Figures S7D,E, 6H and S8A–C). Similarly, serum levels of AST and ALT in the 1/2‐dose EV‐AAV‐LDLR group were restored to normal levels suggesting a substantial recovery of liver function (Figure S8D,E). Additionally, Sirius Red, H&E and Masson's staining of the aortic root and liver demonstrated reduced fibrosis in the 1/2 EV‐AAV‐LDLR group, in contrast to the AAV‐LDLR group which showed no apparent change (Figure S9A,B). Furthermore, the 1/2 EV‐AAV‐LDLR group also showed a significant reduction in both F4/80 and α‐SMA expression in the aortic root. In contrast, the AAV‐LDLR group exhibited only a slight reduction in F4/80 and no significant change in α‐SMA (Figure S10A–C).

These data demonstrate that a half‐dose EV‐AAV‐LDLR maintains therapeutic efficacy in the presence of pre‐existing immunity. By successfully evading neutralizing antibodies, it achieves robust transgene expression comparable to that seen in immunonaive cohorts.

### 3.8 EV‐AAVs Demonstrates Superior Safety Profile With Reduced Liver Damage

**FIGURE 7:**
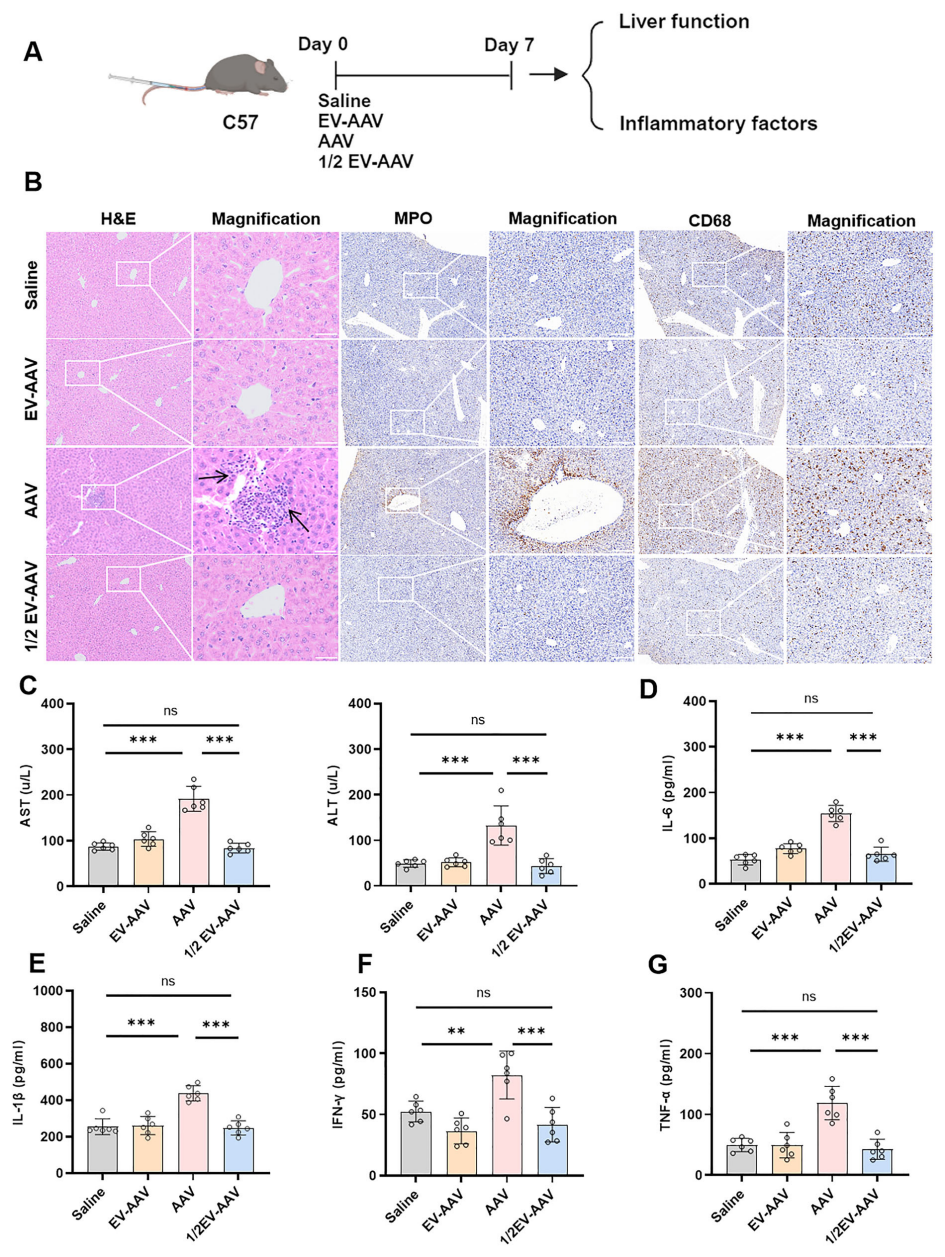
EV‐AAV demonstrates superior safety profile with reduced liver damage compared to AAV. (A) Experimental design schematic. (B) H&E, MPO, CD68 staining to assess liver cell morphology and inflammatory cell infiltration in different groups of C57BL/6 mice. (C) Measurement of AST and ALT levels in serum. (D) ELISA‐based quantification of IL‐6 expression levels in serum. (E) ELISA‐based quantification of IL‐1β expression levels in serum. (F) ELISA‐based quantification of IFN‐γ expression levels in serum. (G) ELISA‐based quantification of TNF‐α expression levels in serum. *n* = 6, data were analyzed using two‐way ANOVA. Values are presented as mean ± SEM. ****p < 0.0001, ***p < 0.001, **p < 0.01, ns indicates no significant difference.

To assess the safety of EV‐AAV, we administered high doses of AAV9 (6e12 vg) or EV‐AAV9 (6e12 vg, 3e12 vg) via tail vein injection in C57BL/6 mice. Liver and serum samples were collected 7 days post‐injection to evaluate hepatotoxicity and inflammatory responses (Figure 7A). Histological analysis by H&E staining revealed substantial infiltration of inflammatory cells in the AAV9‐treated group, while the EV‐AAV9 and 1/2‐dose EV‐AAV9 groups exhibited fewer inflammatory cells and preserved normal hepatocyte morphology (Figure 7B). MPO and CD68 staining further confirmed reduced immune cell infiltration in the EV‐AAV9 groups (Figure 7B).

Additionally, while administration of high‐dose AAV9 caused a dramatic increase in serum AST and ALT levels, the EV‐AAV9 group maintained levels similar to those of the saline control group (Figure 7C). Furthermore, levels of pro‐inflammatory cytokines, including IL‐6, IFN‐γ, IL‐1β and TNF‐α, were significantly elevated in the AAV9 group, whereas no significant changes were observed in the EV‐AAV9‐treated animals (Figure 7D‐G). These findings demonstrate that EV‐AAV9 reduces hepatotoxicity and exhibits lower propensity for inducing cytokine storm compared to AAV9 when administrated at equivalent doses, although it remains to be determined whether these benefits will translate to humans.

## 4 Discussion

The development of EV‐AAV as a gene therapy delivery vector has attracted considerable attention from researchers globally. By combining the stable expression profile of AAV with the EV's shielding capacity, which allow it to bypass the host's pre‐existing anti‐AAV immunity, EV‐AAVs are theoretically positioned to be the next generation of gene therapy vectors, a potential that has been demonstrated by previous studies showing great therapeutic promise in diverse diseases, including cardiovascular, muscular and neurological disorders (Li et al. 2023; Gyorgy et al. 2017; Gyorgy et al. 2014; Schiller et al. 2018).

Although both AAV and EVs can be individually manufactured at large scale, achieving efficient large‐scale production of EV‐AAV complexes remains a major technical challenge. This difficulty arises because EV‐AAVs are generated only as a natural byproduct during the AAV packaging process, and previous studies have therefore concentrated primarily on their isolation and purification technique development rather than on enhancing their yield. Building on this rationale, in the present study we sought to leverage the CNP technique to actively promote EV‐AAV production beyond the limited levels achieved through natural byproduct formation.

By inducing localized plasma membrane disruption, which in turn elevates HSPs and intracellular calcium levels, the CNP technique has been shown in our previous work to markedly enhance EV yield and improve mRNA encapsulation efficiency (Yang et al. 2020; You et al. 2025; You et al. 2023). Building on this foundation, we applied the CNP approach to EV‐AAV manufacturing and found that it achieved an order‐of‐magnitude increase in yield compared to the natural byproduct formation process. We attribute the enhanced EV‐AAV production to CNP‐induced transient membrane disruption. Calcium influx and heat shock protein (HSP) upregulation, then rapid membrane repair and EV shedding. During this restructuring, AAVs are passively co‐encapsulated into nascent EVs without requiring active endosomal recruitment. This technological breakthrough represents a pivotal step forward for the field, as it transforms EV‐AAVs from a scarce and poorly controllable byproduct into a readily scalable vector platform, thereby unlocking their true translational potential in gene therapy. We further characterized the encapsulation pattern of CNP‐generated EV‐AAVs and revealed that the boosted EV yield formed the foundation for EV‐AAV scale‐up, as viruses were passively entrapped within the approximately 11‐fold increase in EVs, accompanied by a nearly 11‐fold enhancement in encapsulation efficiency. While this study successfully established CNP‐based EV‐AAV production platform, our optimization efforts were limited to the timing of CNP application relative to AAV packaging. Looking ahead, there are at least two major avenues to further scale up EV‐AAV production based on CNP platform: (i) fine‐tuning CNP parameters to achieve greater EV yields, and (ii) innovating AAV packaging strategies to enhance viral generation.

Consistent with previous findings, EV‐AAV was delivered more efficiently than AAV when administered at the same dose (Li et al. 2023). This enhanced delivery can be attributed to the bilayer membrane structure of EV‐AAVs, which protects the viral cargo from degradation and pre‐existing neutralizing antibodies (NAbs) in vivo, ensuring the vector effectively reaches its target cells (Doyle and Wang 2019; Meliani et al. 2017; Oliveira et al. 2024; Sul et al. 2024; van Niel et al. 2018; Gurung et al. 2021; Joshi et al. 2020). Notably, while previous studies on EV‐AAVs have primarily focused on their application in the cardiac or central nervous systems, our study highlights a unique advantage: their exceptional suitability for liver‐targeted gene therapy (Yao et al. 2021; Zhang et al. 2020; van der Koog et al. 2022). This is largely attributed to the natural tropism of EVs for the liver, a property that ensures a high rate of internalization by hepatocytes. This inherent affinity allowed EV‐AAVs to outperform traditional AAVs in treating the Familial Hypercholesterolaemia model, making them an exceptionally promising vector for a wide range of hepatic gene therapy applications.

While previous studies have highlighted the promise of EV‐AAVs, there has been a lack of systematic evaluation regarding the therapeutic efficacy of low‐dose formulations (You et al. 2023; Doyle and Wang 2019; Meliani et al. 2017; Oliveira et al. 2024). Our study is the first to address this gap, presenting the initial application of CNP‐generated EV‐AAV vectors for gene replacement therapy in a mouse model of Familial Hypercholesterolaemia. Through a systematic assessment of both therapeutic efficacy and biological safety, we demonstrated that a half‐dose of EV‐AAV‐LDLR achieved efficacy equivalent to a full dose of conventional AAV, while introducing minimal hepatic toxicity and inflammatory responses, which are significant limitations of conventional AAV therapy. More importantly, half‐dose EV‐AAV‐LDLR also showed superior therapeutic efficacy over AAV in the presence of pre‐existing neutralizing antibodies, which we attribute to the EVs' shielding capacity. This finding carries profound implications for clinical translation, as it not only demonstrates a path to reducing the cost and off‐target toxicity of gene therapy but also paves the way for a more accessible and safer treatment paradigm.

In summary our study successfully established a CNP‐produced EV‐AAV platform, which addresses the critical challenge of scaling up EV‐AAV production. This work paves the way for the translational potential of EV‐AAV in gene therapy. In addition, by systematically assessing the therapeutic effect of CNP‐produced EV‐AAV‐LDLR in a LDLR^−/−^ FH mouse model, we confirmed the superior therapeutic efficacy and biological safety of a lower dose of EV‐AAV, and notably, it remains highly effective in the presence of pre‐existing immunity. These findings collectively highlight the cost‐effectiveness of EV‐AAV which represents a major advantage for its future use in gene therapy for a broad spectrum of genetic disorders characterized by lipid metabolism dysregulation and beyond.

## Author Contributions

**Yuting Yan**: writing – original draft, methodology, conceptualization, data curation, formal analysis, validation. **Yi You**: conceptualization, validation, methodology, writing – original draft. **Shuhong Ma**: conceptualization, writing – original draft, validation, methodology, funding acquisition. **Hui Yi**: writing – review and editing, validation, methodology, conceptualization. **Guangduo Chen**: methodology, writing – review and editing. **Jie Ni**: validation, formal analysis. **Changyan Chen**: validation. **Wenyu Ke**: validation. **Lingying Li**: validation. **Rui Bai**: validation. **Yuqing Ran**: validation. **Wenjing Lu**: validation. **Min Zhu**: validation. **Yongshuai Zhang**: validation. **Jing Dai**: validation. **Man Qi**: validation. **Feng Lan**: funding acquisition, conceptualization. **Andrew Lee**: methodology, writing – original draft, investigation. **Ran Zhang**: writing – review and editing, visualization, methodology, funding acquisition. **Xujie Liu**: funding acquisition, writing – review and editing, writing – original draft, methodology. **Zhaoyang Chen**: writing – review and editing, writing – original draft, funding acquisition, investigation, methodology, conceptualization.

## Funding

We gratefully acknowledge funding support from the National Key Research and Development Program of China (2021YFC2701703, 2023YFA0915002), National Natural Science Foundation of China (NSFC) General Program (82470370, 82400587, 82400381, 82350610278), CAMS Innovation Fund for Medical Sciences (CIFMS) (2021‐RC310‐012, 2022‐I2M‐2‐001, 2023‐I2M‐1‐003, 2023‐I2M‐2‐003 and 2024‐I2M‐ZH‐002), National High‐Level Hospital Clinical Research Funding (2022‐GSP‐GG‐7, 2024GZZD‐02), Non‐profit Central Research Institute Fund of Chinese Academy of Medical Sciences (2019PT320026), Shenzhen Medical Research Fund (B2302048), Shenzhen Fundamental Research Program (ZDSYS20200923172000001, JCYJ20220531091615034), Shenzhen High‐level Hospital Construction Fund (GSP‐ZDSYS‐020), National Clinical Research Center for Geriatric Diseases, Chinese PLA General Hospital (NCRCG‐PLAGH‐2024008), China Postdoctoral Science Foundation (grant 2024T170070) and Youth Talent Support Program of Fujian Province (Eyas Plan of Fujian Province 2022), Shenzhen Bay Laboratory Proof‐of‐Concept Grants (S231801003)and Excellent Young Scholars Cultivation Project of Fujian Medical University Union Hospital (2022XH020).

## Conflicts of Interest

The authors declare no conflicts of interest.

## Supporting information




**Supplementary Figures**: jev270186‐sup‐0001‐figureS1‐S10.docx

## Data Availability

The data that support the findings of this study are available from the corresponding author upon reasonable request.
