# Supplementary material for: Efficient Scaling up EV‐AAVs Production via Cellular Nanoporation for Familial Hypercholesterolaemia Therapy
Source: J Extracell Vesicles. 2025 Nov 11;14(11):e70186. doi: 10.1002/jev2.70186 (PMC12603781; doi:10.1002/jev2.70186)
Supplement: Supplementary file 1 — Supplementary Figures: jev270186‐sup‐0001‐figureS1‐S10.docx [file JEV2-14-e70186-s001.docx]

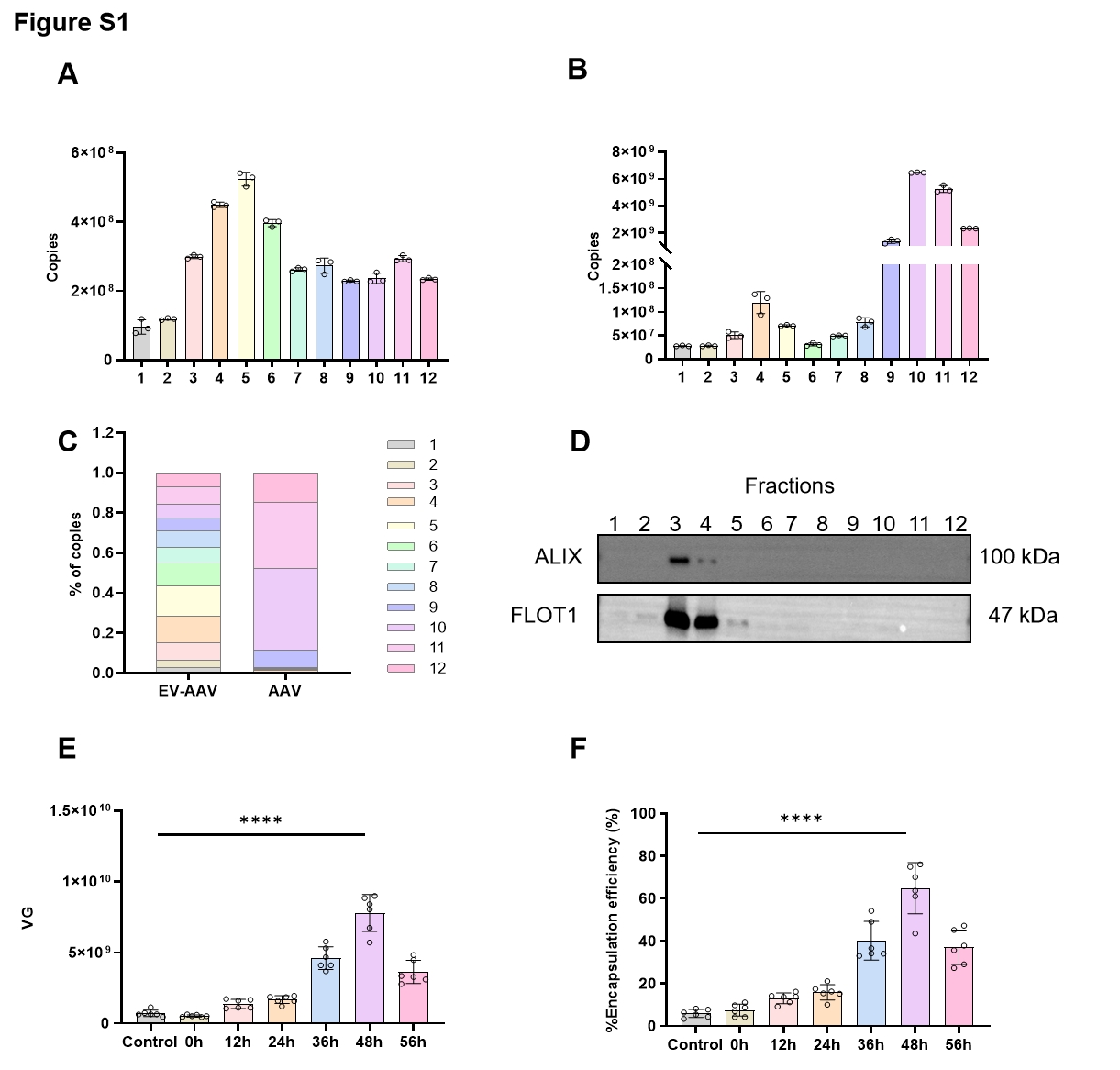


**Figure S1. Extraction and purification of EV-AAV.** (A) qPCR was performed to measure the AAV genome content in 12 fractions of EV-AAV samples purified by iodixanol gradient. n=3. (B) qPCR was also used to detect the AAV genome content in 12 fractions of free AAV samples purified by iodixanol gradient. n=3. (C) Percentage of AAV genomes in each fraction of EV-AAV and AAV. (D) Western blot analysis was conducted to assess the protein expression of Alix and Flot1 in the 12 fractions of EV-AAV. (E) The total amount of purified EV-AAV produced after CNP at different time points. n=6, data were analyzed using two-way ANOVA. Values are presented as mean ± SEM. ****P<0.0001. (F) The encapsulation efficiency of EV-AAV was monitored at various time points (0, 12, 24, 36, 48, and 56 h) following CNP treatment. n=6, data were analyzed using two-way ANOVA. Values are presented as mean ± SEM. ****P<0.0001.


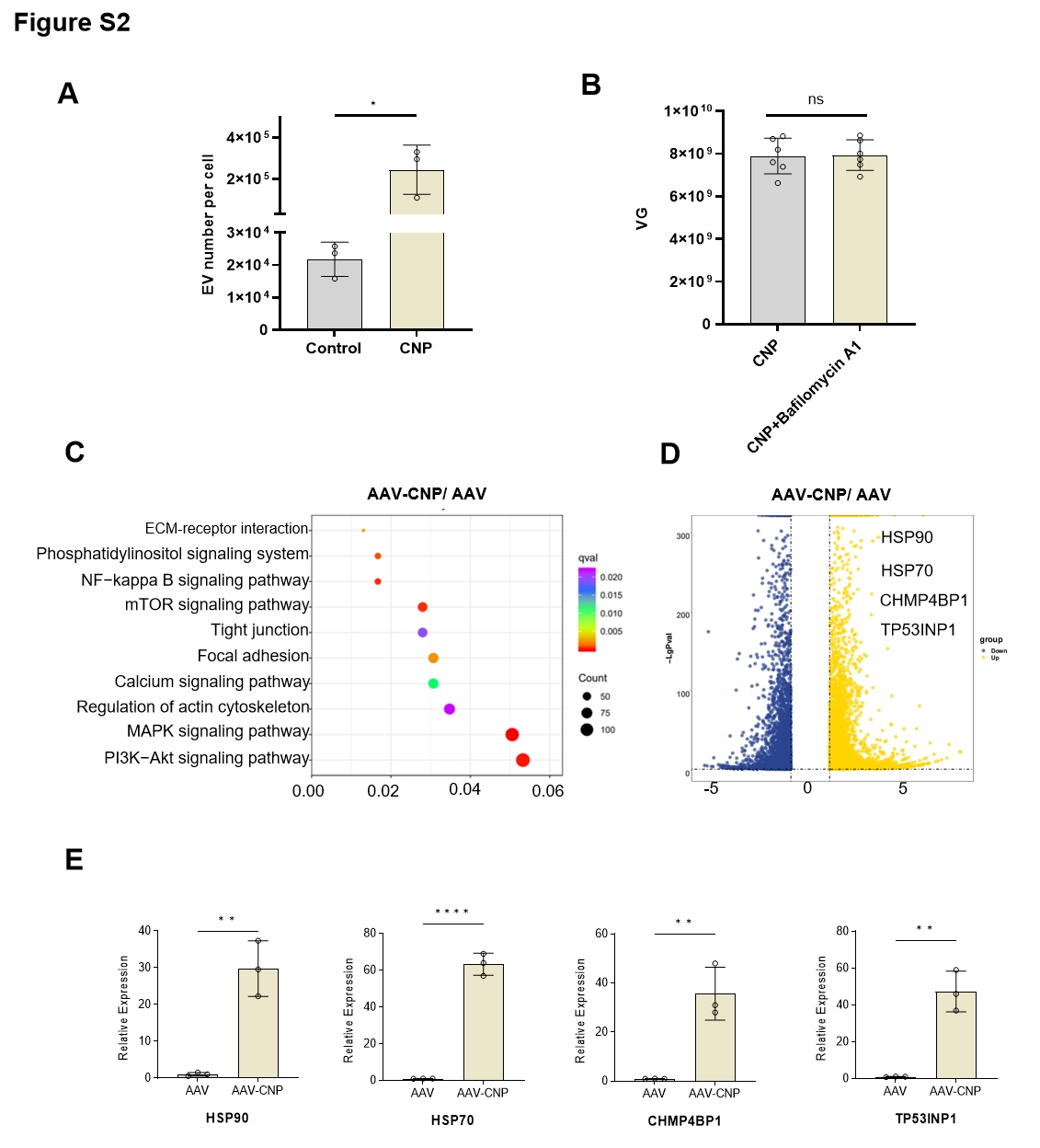


**Figure S2. CNP activates HSP proteins and calmodulin to induce passive EV-AAV encapsulation.** (A) EV quantification by nanoparticle tracking analysis (NTA) in supernatants of HEK293T cells treated with or without CNP for 24 h. (B) EV quantification by NTA in cells pretreated with Bafilomycin A1 (Baf-A1, 100 nM) for 2 h prior to CNP stimulation (24 h). (C) Transcriptome sequencing of HEK293T cells pre-loaded with AAV for 48 h, followed by ± CNP treatment for 24 h. Differential gene expression analysis included KEGG pathway enrichment and volcano plots. (D) qPCR validation of HSP90, HSP70, CHMP4B, and TP53INP1 expression in AAV-preloaded HEK293T cells under ± CNP conditions (24 h). Data represent mean ± SEM (n=3). Statistical significance was determined by two-way ANOVA with Bonferroni post-test: **P < 0.01,****P < 0.0001.


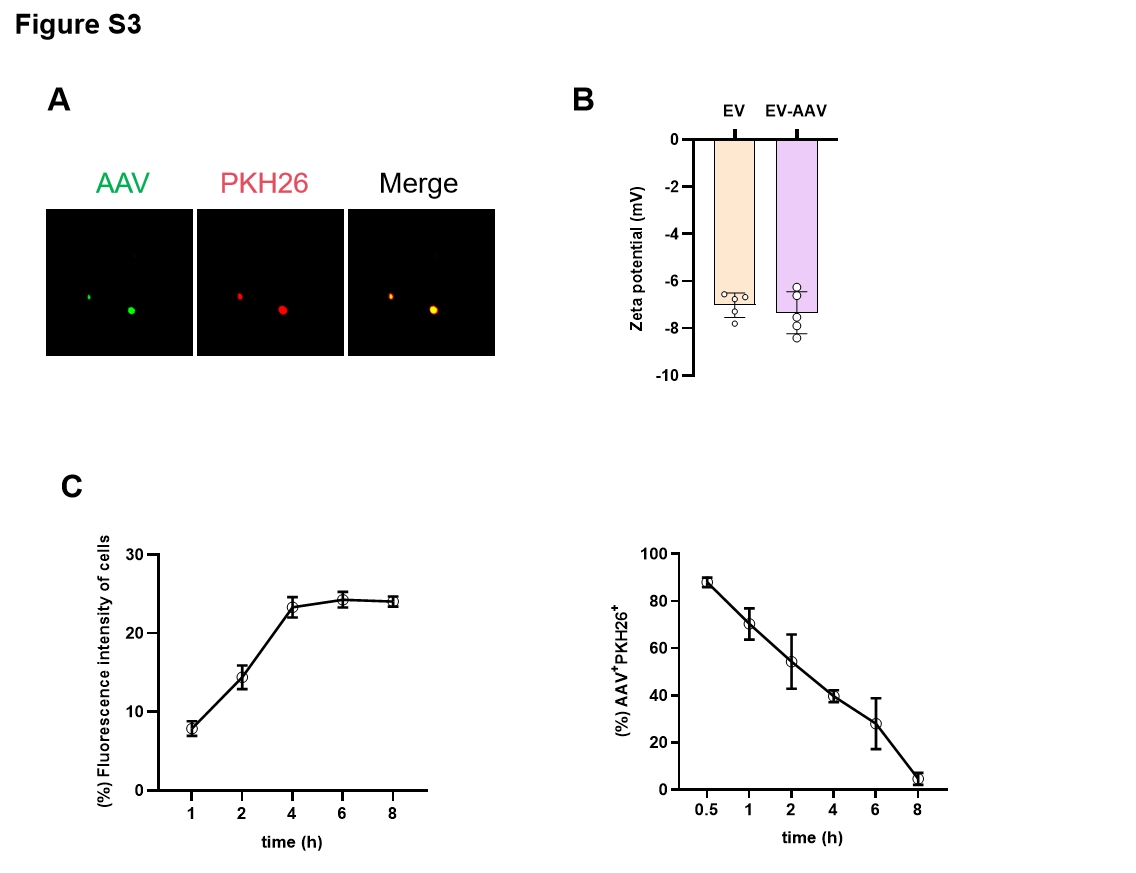


**Figure S3. Quality Control Testing for EV-AAV.** (A) Immunofluorescence was performed to detect the co-localization of AAV and PKH26. (B) ZetaView was used to measure the zeta potential levels of EV and EV-AAV (n=5). (C) The fluorescence intensity of AAV in Figure 1H was quantified using ImageJ software(left), The percentage of PKH26 and AAV double-positive particles (right).


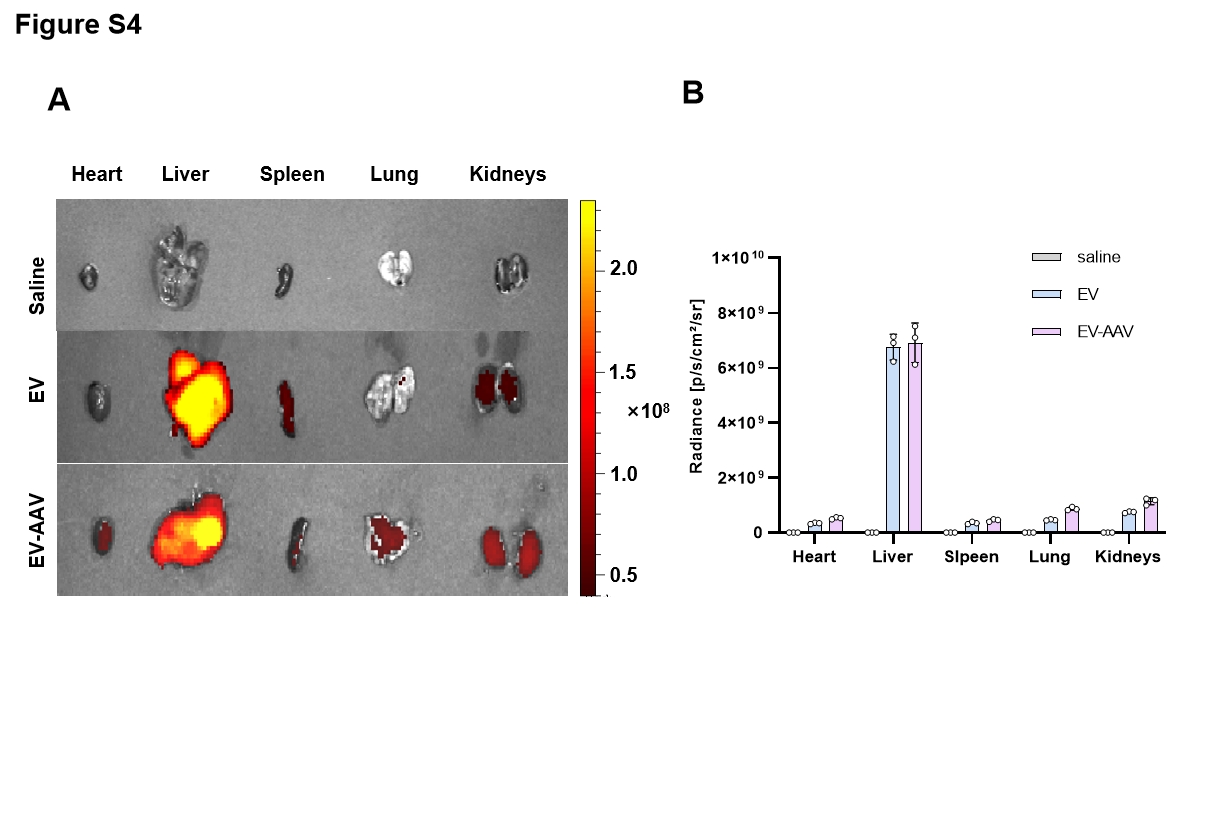


**Figure S4. EVs exhibit hepatic tropism.** (A) C57BL/6 mice (n=3) were intravenously injected via the tail vein with saline (control), PHK26-labeled EV-AAV and EVs. At 24 h post-injection, mice were euthanized, and major organs (heart, liver, spleen, lung, kidney) were excised for ex vivo fluorescence quantification using an IVIS imaging system. (B) Biodistribution analysis revealed that both EV-AAV and EVs predominantly accumulated in the liver, with significantly enhanced hepatic targeting compared to peripheral organs.


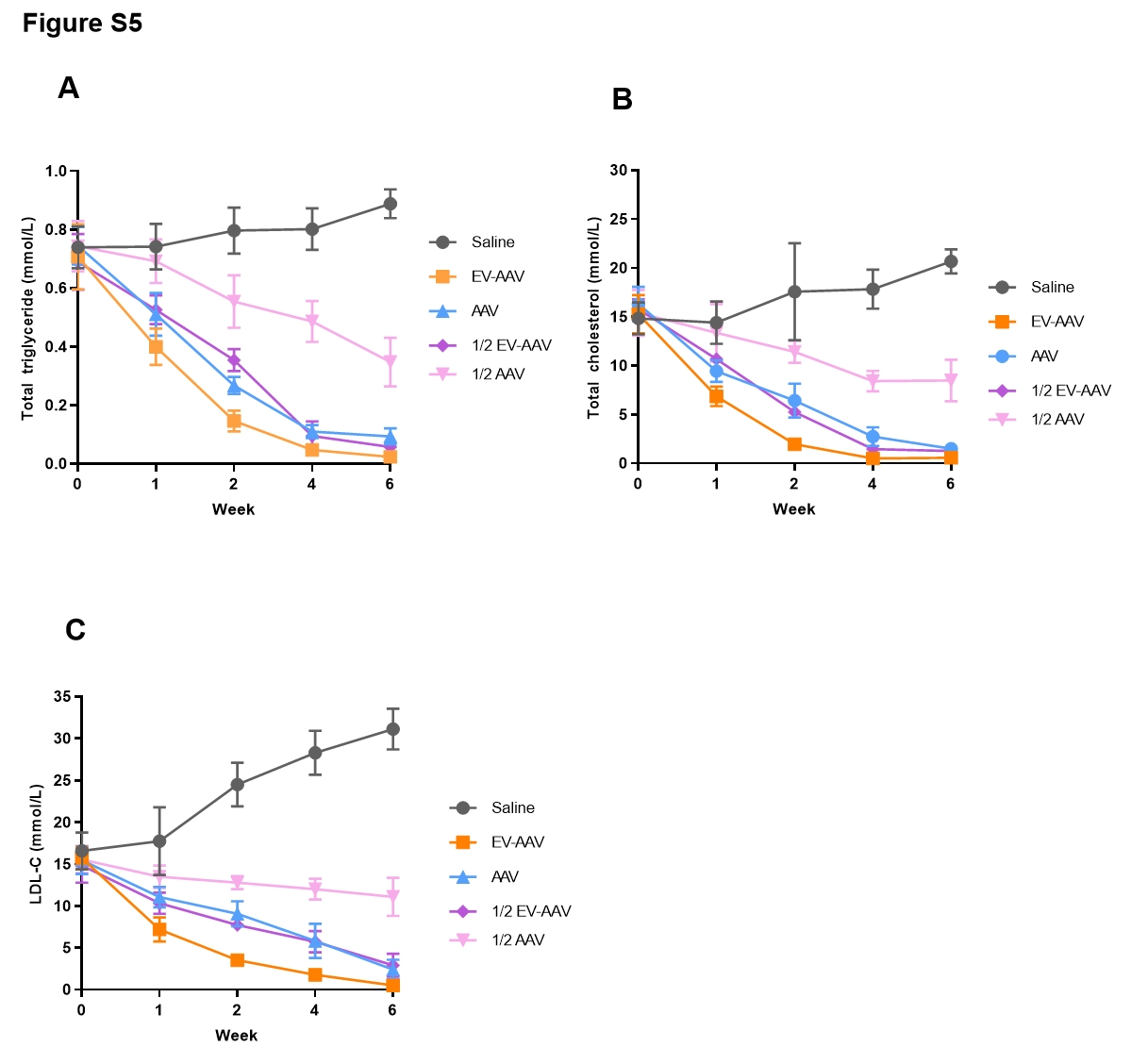


**Figure S5. EV-AAV treatment resulted in the normalization of lipid profiles.** (A-C) Experimental design: LDLR^-/-^ mice were either fed a Western diet for 12 weeks or not, followed by tail vein injection of saline, EV-AAV-LDLR (1.2e11 vg, 6e10 vg), AAV-LDLR (1.2e11 vg, 6e10 vg). Serum levels of total triglycerides, total cholesterol, and LDL-C were measured at 0 h, 1 week, 2 weeks, 4 weeks, and 6 weeks post-injection.


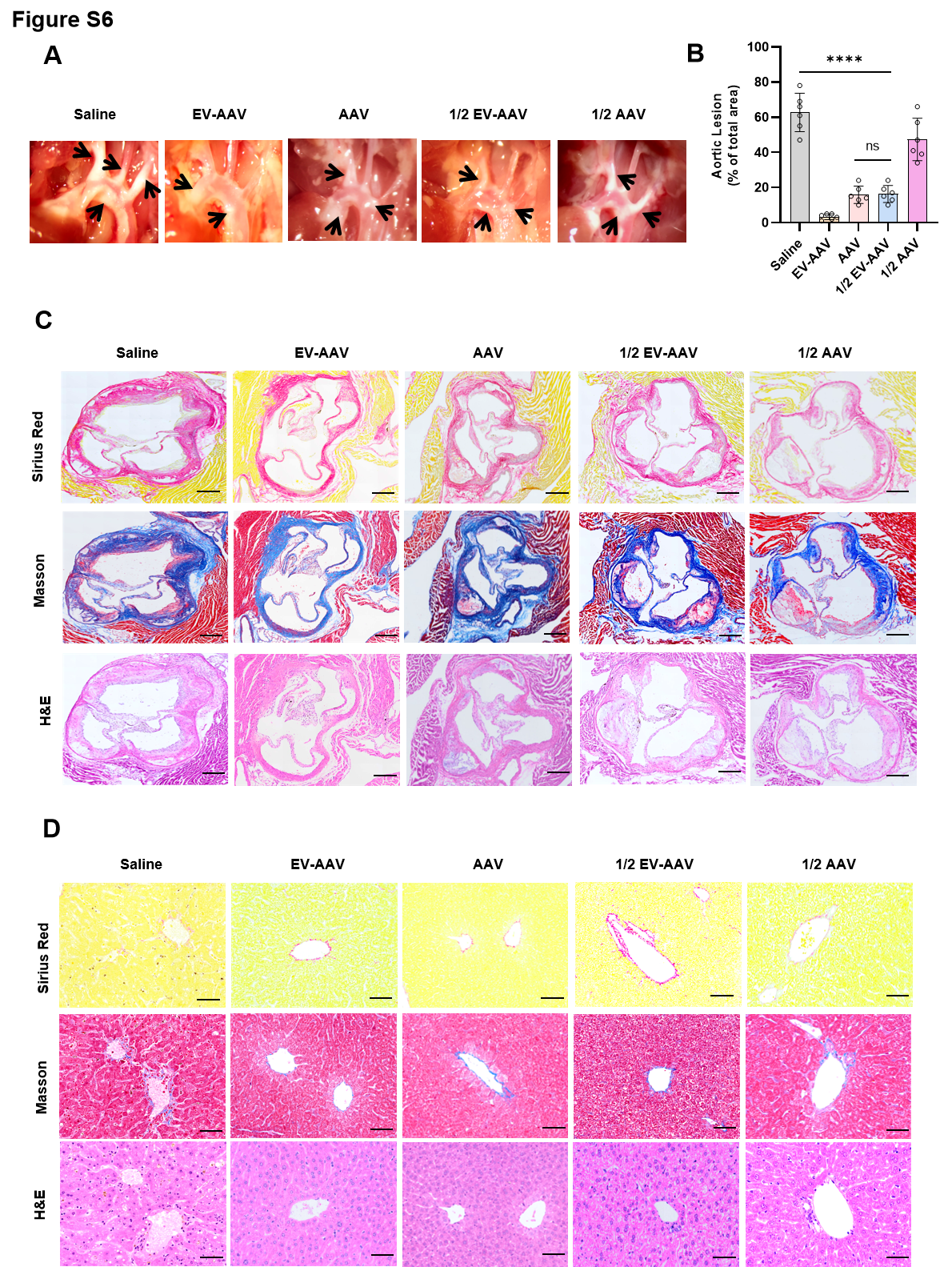


**Figure S6. EV-AAV treatment reduces fibrosis in FH mice.** (A) Experimental design: LDLR^-/-^ mice were either fed a Western diet for 12 weeks or not, followed by tail vein injection of saline, EV-AAV-LDLR (1.2e11 vg, 6e10 vg), AAV-LDLR (1.2e11 vg, 6e10 vg). Six weeks post-injection, aortic morphology was assessed. Black arrows indicate plaque areas. (B) Plaque area was quantified using ImageJ. (C) Aortic root tissue was collected and cryosectioned, followed by H&E, Masson’s trichrome, and Sirius Red staining. Scale bar = 200 μm. (D) Liver tissue was also cryosectioned and stained with H&E, Masson’s trichrome, and Sirius Red. Scale bar = 50 μm. n=6; data were analyzed using two-way ANOVA. Values are presented as mean ± SEM. ****P<0.0001.


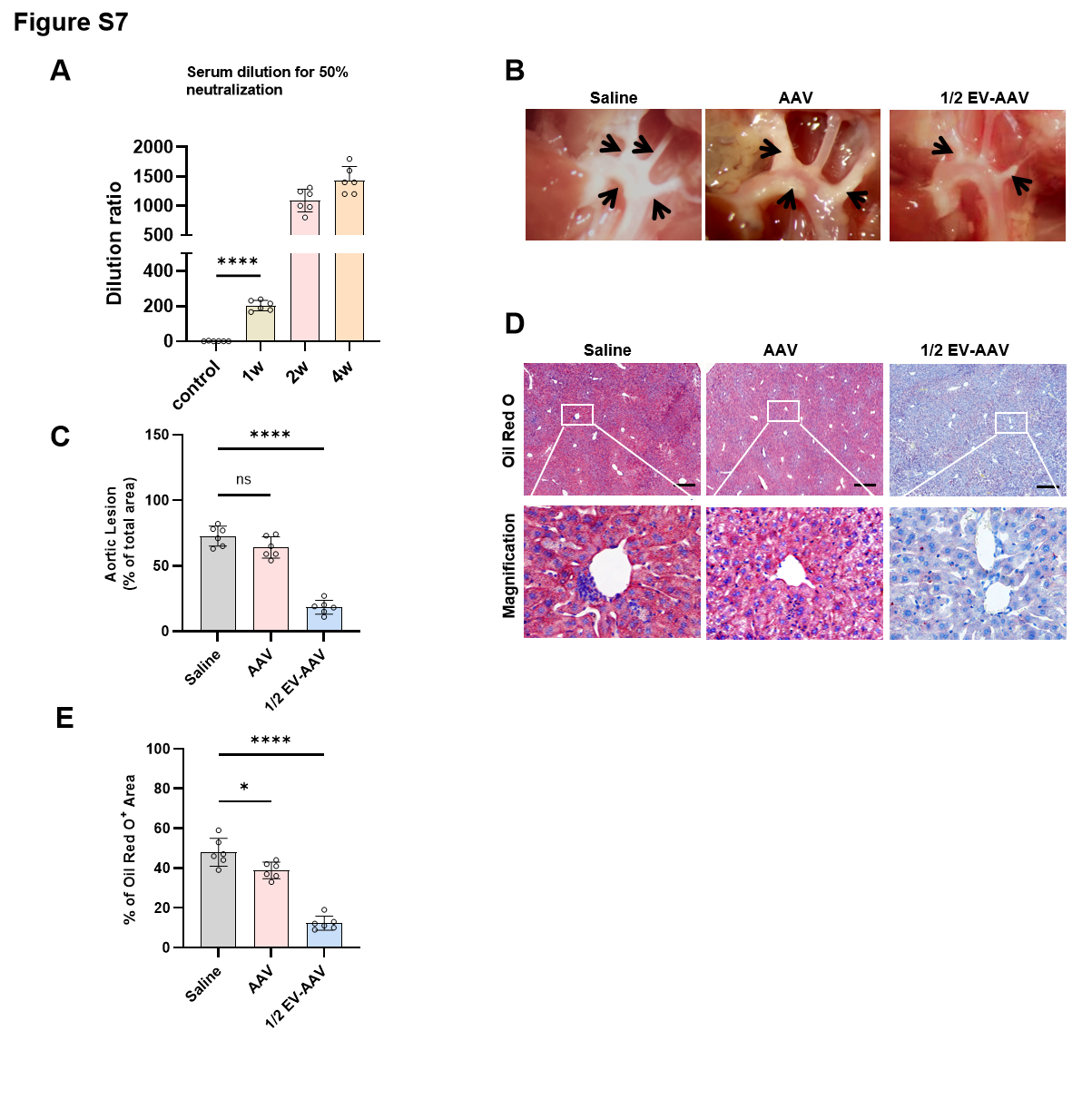


**Figure S7. Second injection of EV-AAV treatment in FH mice still has an effect.** (A) LDLR^-/-^ mice were fed a Western diet for 11 weeks, followed by tail vein injection of AAV9 (1.2e11 vg). Serum was collected 1, 2, and 4 weeks after injection for neutralizing antibody testing. (B) LDLR^-/-^ mice were fed a Western diet for 11 weeks, followed by tail vein injection of AAV9 (1.2e11 vg). After 1 week of continued Western diet feeding, mice were injected with saline, AAV-LDLR (1.2e11 vg), or EV-AAV-LDLR (6e10 vg). Six weeks post-injection, aortic morphology was assessed. Black arrows indicate plaque areas. (C) Plaque area was quantified using ImageJ. （D） Representative images of Oil Red O staining in liver tissue. Scale bar = 50μm. (E) Quantification of lipid deposition in liver tissue using ImageJ. n=6; data were analyzed using two-way ANOVA. Values are presented as mean ± SEM. ****P<0.0001.


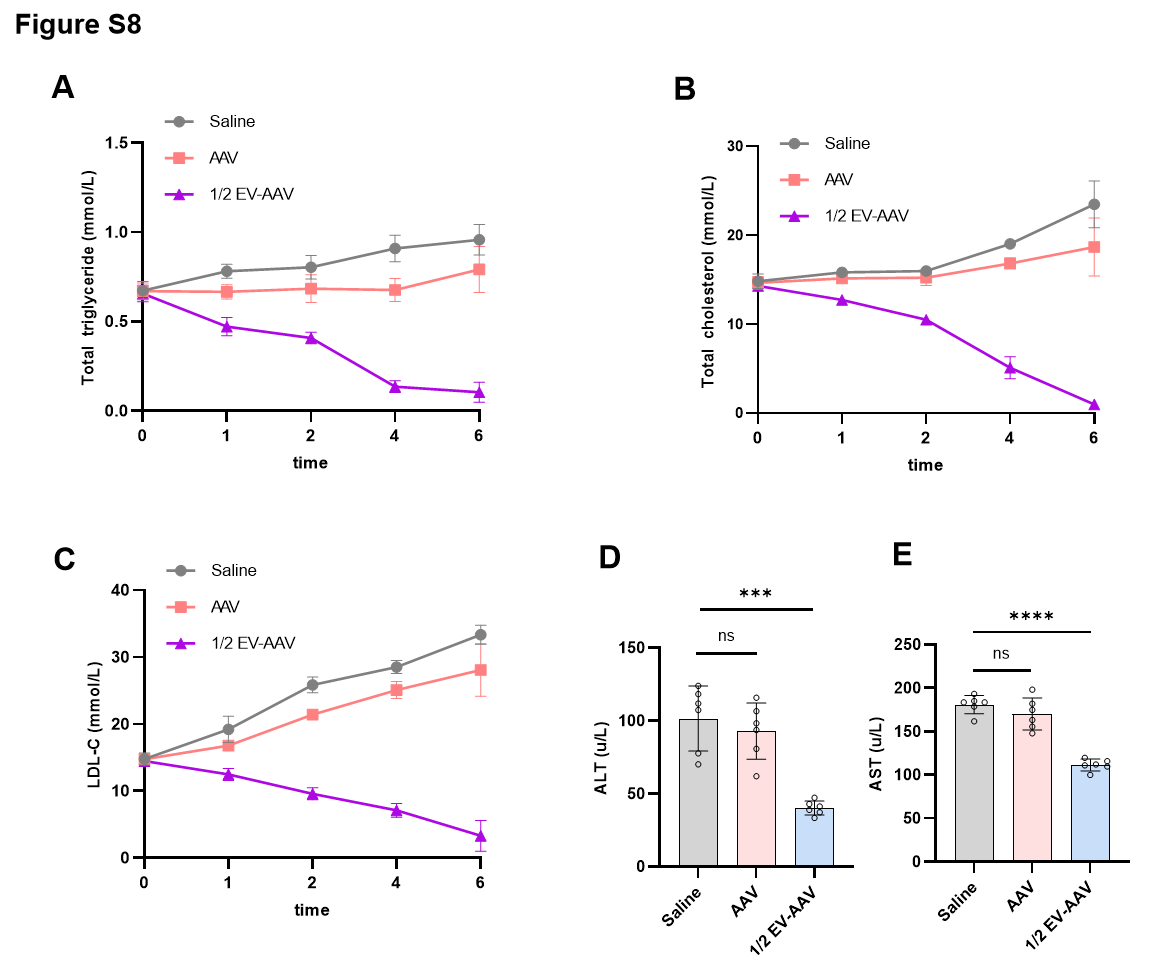


**Figure S8. Second injection of EV-AAV restored lipid levels and liver function.** (A-C) LDLR^-/-^ mice were fed a Western diet for 11 weeks, followed by tail vein injection of AAV9 (1.2e11 vg). After 1 week of continued Western diet feeding, mice were injected with saline, AAV-LDLR (1.2e11 vg), or EV-AAV-LDLR (6e10 vg). Serum levels of total triglycerides, total cholesterol, and LDL-C were measured at 0 h, 1 week, 2 weeks, 4 weeks, and 6 weeks post-injection. （D-E）Analysis of serum AST and ALT levels in mice. N=6, data were analyzed using two-way ANOVA. Values are presented as mean ± SEM. ****P<0.0001, ***P<0.001, ns indicates no significant difference.


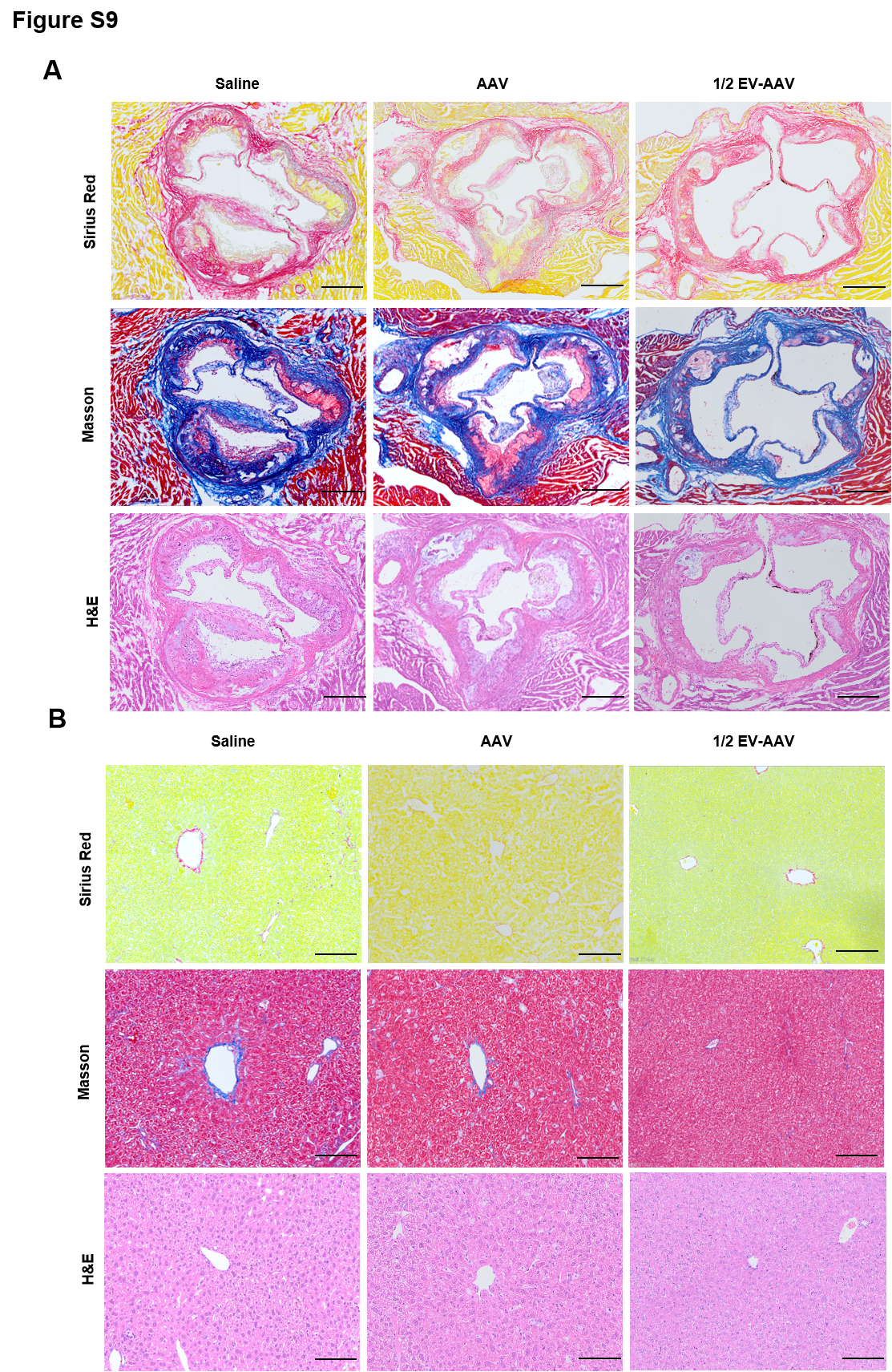


**Figure S9. Second injection of EV-AAV treatment reduces fibrosis in FH mice.** (A) LDLR^-/-^ mice were fed a Western diet for 11 weeks, followed by tail vein injection of AAV9 (1.2e11 vg). After 1 week of continued Western diet feeding, mice were injected with saline, AAV-LDLR (1.2e11 vg), or EV-AAV-LDLR (6e10 vg). Aortic root tissue was collected and cryosectioned 6 weeks post-injection, followed by H&E, Masson’s trichrome, and Sirius Red staining. Scale bar = 200 μm. (D) Liver tissue was also cryosectioned and stained with H&E, Masson’s trichrome, and Sirius Red. Scale bar = 50 μm.

**
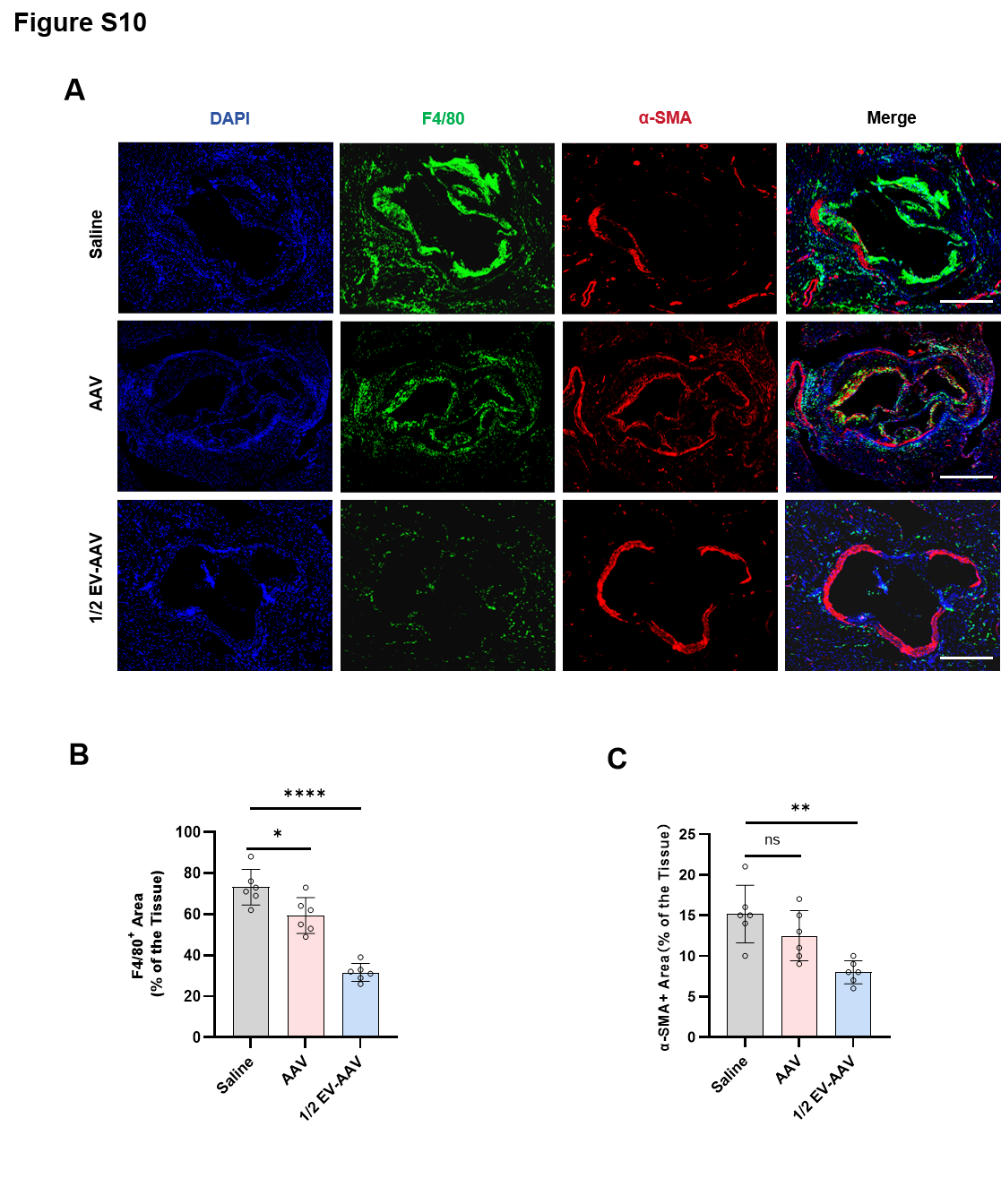
**

**Figure S10. Second injection of EV-AAV treatment inhibited plaque formation.** (A) LDLR^-/-^ mice were fed a Western diet for 11 weeks, followed by tail vein injection of AAV9 (1.2e11 vg). After 1 week of continued Western diet feeding, mice were injected with saline, AAV-LDLR (1.2e11 vg), or EV-AAV-LDLR (6e10 vg). Immunofluorescence analysis of α-SMA and F4/80 expression and distribution in the aortic valve at 6 weeks post-injection. Scale bar = 200 μm. (B) ImageJ analysis of α-SMA and F4/80 expression levels. N=6, data were analyzed using two-way ANOVA. Values are presented as mean ± SEM. ****P<0.0001, **P <0.01, ns indicates no significant difference.
